# Ablation of PGC-1β Results in Defective Mitochondrial Activity, Thermogenesis, Hepatic Function, and Cardiac Performance

**DOI:** 10.1371/journal.pbio.0040369

**Published:** 2006-11-07

**Authors:** Christopher J Lelliott, Gema Medina-Gomez, Natasa Petrovic, Adrienn Kis, Helena M Feldmann, Mikael Bjursell, Nadeene Parker, Keira Curtis, Mark Campbell, Ping Hu, Dongfang Zhang, Sheldon E Litwin, Vlad G Zaha, Kimberly T Fountain, Sihem Boudina, Mercedes Jimenez-Linan, Margaret Blount, Miguel Lopez, Aline Meirhaeghe, Mohammad Bohlooly-Y, Leonard Storlien, Maria Strömstedt, Michael Snaith, Matej Orešič, E. Dale Abel, Barbara Cannon, Antonio Vidal-Puig

**Affiliations:** 1 Department of Clinical Biochemistry, University of Cambridge, Cambridge, United Kingdom; 2 AstraZeneca R&D, Mölndal, Sweden; 3 The Wenner-Gren Institute, Stockholm University, Stockholm, Sweden; 4 Division of Cardiology, University of Utah, Salt Lake City, Utah, United States of America; 5 Division of Endocrinology, Metabolism, and Diabetes and Program in Human Molecular Biology and Genetics, University of Utah, Salt Lake City, Utah, United States of America; 6 INSERM, Institut Pasteur de Lille, Lille Cedex, France; 7 VTT Technical Research Centre of Finland, Espoo, Finland; Stanford University School of Medicine, United States of America

## Abstract

The transcriptional coactivator peroxisome proliferator-activated receptor-gamma coactivator-1β (PGC-1β) has been implicated in important metabolic processes. A mouse lacking PGC-1β (PGC1βKO) was generated and phenotyped using physiological, molecular, and bioinformatic approaches. PGC1βKO mice are generally viable and metabolically healthy. Using systems biology, we identified a general defect in the expression of genes involved in mitochondrial function and, specifically, the electron transport chain. This defect correlated with reduced mitochondrial volume fraction in soleus muscle and heart, but not brown adipose tissue (BAT). Under ambient temperature conditions, PGC-1β ablation was partially compensated by up-regulation of PGC-1α in BAT and white adipose tissue (WAT) that lead to increased thermogenesis, reduced body weight, and reduced fat mass. Despite their decreased fat mass, PGC1βKO mice had hypertrophic adipocytes in WAT. The thermogenic role of PGC-1β was identified in thermoneutral and cold-adapted conditions by inadequate responses to norepinephrine injection. Furthermore, PGC1βKO hearts showed a blunted chronotropic response to dobutamine stimulation, and isolated soleus muscle fibres from PGC1βKO mice have impaired mitochondrial function. Lack of PGC-1β also impaired hepatic lipid metabolism in response to acute high fat dietary loads, resulting in hepatic steatosis and reduced lipoprotein-associated triglyceride and cholesterol content. Altogether, our data suggest that PGC-1β plays a general role in controlling basal mitochondrial function and also participates in tissue-specific adaptive responses during metabolic stress.

## Abstract 2

The authors conduct an in-depth analysis of a PGC-1β knockout mouse; these animals posses specific defects in basal mitochondrial function and adaptation to metabolic stress.

## Introduction

Transcriptional coactivators (TCs) have emerged as key proteins that are capable of regulating entire metabolic networks [1]. TCs are often selective with respect to the repertoire of transcription factor partners. The mechanisms used to modulate transcription may vary according to the specific TC, their transcription factor partners, and the nucleic acid environment in which they operate. TCs provide an additional layer of control by facilitating temporal and tissue-specific regulation of metabolic pathways [1].

Peroxisome proliferator-activated receptor-gamma coactivator-1α (PGC-1α, previously known as PGC-1) [2] is involved in coordinating diverse organ-specific transcription programs. For example, PGC-1α not only activates the thermogenic program in brown adipose tissue (BAT) in response to environmental cold exposure but also modulates the metabolic transition from the fed to the fasted state in the liver [2–4]. Two other PGC-1α-related genes have subsequently been found, PGC-1-related coactivator (PRC) [5] and PGC-1β (PERC/ERRL-1) [6–8]. All three family members share a similar basic structure; an N-terminal domain containing nuclear hormone receptor–interacting motifs (the LXXLL motifs) and a C-terminal region containing RNA-binding motifs (RMM) and serine-arginine rich (RS) domains. Whereas PRC is primarily an activator of nuclear respiratory factor-1 [5], PGC-1α and PGC-1β have a broader repertoire of partners [7,9–12].

The gene expression level of PRC is conserved between tissues [5], but the highest expression of PGC-1α and PGC-1β is found in oxidative tissues, including BAT, heart, and skeletal muscle [2,6,7]. However, despite this overlap of expression pattern, PGC-1α and −1β display different responses to physiological stimuli. Whereas PGC-1α is transcriptionally up-regulated in BAT and skeletal muscle during cold exposure, PGC-1β expression remains stable under these conditions [2,6,7]. Furthermore, up-regulation of PGC-1α is essential for a normal response to acute cold exposure, indicating that PGC-1β is not able to compensate for all aspects of a lack of PGC-1α [13,14]. Similarly PGC-1α is up-regulated in liver in response to fasting, whereas PGC-1β expression remained stable [6,7,9], further suggesting that PGC-1α is more often regulated transcriptionally than PGC-1β.

Overexpression studies both in vivo and in vitro have revealed that both PGC-1α and −1β are capable of stimulating mitochondrial biogenesis, increasing mitochondrial oxygen consumption, and elevating gene expression of fatty acid oxidation and mitochondrial electron transport chain (ETC) components [7,9,15]. In addition, bioenergetic studies of C2C12 myotubes demonstrated that overexpression of PGC-1α or −1β enhances mitochondrial proton leak [15]. Thus PGC-1β increases mitochondrial energy production through a combination of activated fatty acid oxidation gene expression with increased mitochondrial activity. A role for PGC-1β enhancing mitochondrial activity is further supported by the fact that transgenic overexpression of PGC-1β in mice results in elevated energy expenditure, resistance to high-fat diet (HFD), and genetically induced obesity [10].

Despite their functional similarities, it has recently been suggested that PGC-1α and −1β may have differing metabolic roles in liver. PGC-1α stimulates expression of gluconeogenic genes both in vivo and in vitro [3,9,13,16], and based on data from mice exposed to a diet enriched in saturated fat for a short period of time, it appears that PGC-1β but not PGC-1α interacts with SREBP and LXRα to enhance hepatic lipogenesis and lipoprotein secretion [11]. In addition, PGC-1β interacts with Foxa2 to facilitate hepatic secretion of very low density lipoprotein (VLDL) [17], suggesting a central role for PGC-1β in hepatic lipid metabolism. The second organ where PGC-1α and −1β are also highly expressed is the heart [2,6], but only the role of PGC-1α in cardiac energy metabolism has been studied in detail. Overexpression of PGC-1α in cardiac muscle in vivo leads to uncontrolled mitochondrial proliferation, leading to cardiomyopathy and congestive heart failure due to contractile dysfunction [18,19]. Similarly, PGC-1α knockout mice also develop heart failure with abnormal heart rate and impaired ventricular function [14,20], again suggesting that despite their similar mode of action, PGC-1β is unable to compensate fully for a lack of PGC-1α.

To investigate the function of PGC-1β, we have generated and phenotyped a mouse model lacking PGC-1β (the PGC1βKO mouse). Using a combination of physiological experiments guided by a systems biology approach, we showed that PGC-1β plays a general role in regulating the mitochondrial activity in tissues with active oxidative metabolism. In all tissues studied, lack of PGC-1β was associated with a reduction in the expression of mitochondrial genes, including intermediary subunits and enzymes connected to ETC and oxidative phosphorylation (OxPhos). Despite these mitochondrial defects, the PGC1βKO mouse was able to cope when challenged with a range of physiological stressors including cold acclimatisation and adrenergically-mediated BAT and cardiac stimulation but with abnormal compensatory responses. Altogether, these data suggest that PGC-1β is an important regulator of basal energy homeostasis and essential for the proper metabolic tuning in acute stress situations in multiple organs.

## Results

### Generation of a PGC1βKO Mouse

**Figure 1:**
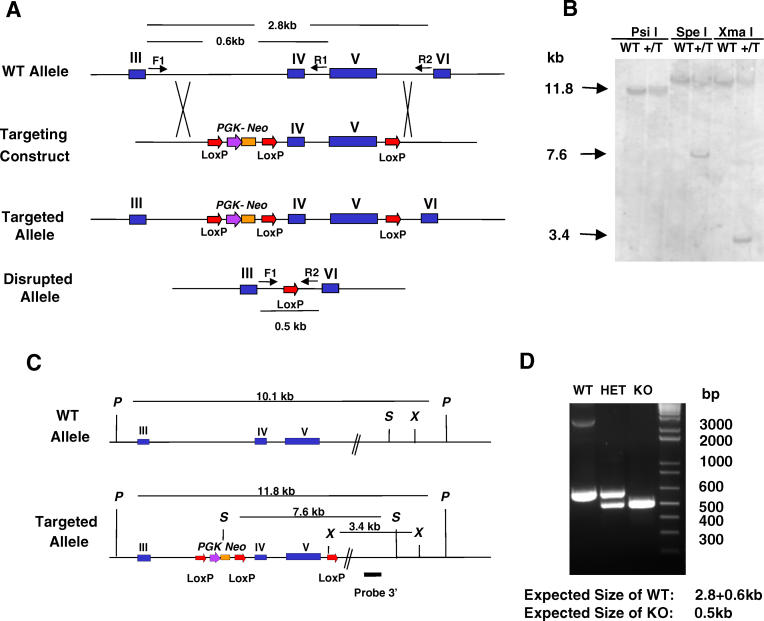
Generation of the PGC1βKO Mouse (A) Design of the targeting construct for the generation of the PGC1βKO mouse using a phosphoglycerate kinase-neomycin phosphotransferase (PGK-Neo)–based LoxP cassette inserted between exons III and IV and a third LoxP site between exons V and VI. Total collapse of the LoxP sites deletes exons 4 and 5 of the *PGC-1*β gene. (B) A Southern blot confirming the generation of the targeted allele in ES cells. WT, wild type ES cells; +/T, ES cells heterozygous for the targeted allele, T. (C) Schematic showing the restriction digest strategy for the southern blot confirming the generation of the targeted allele in ES cells. P, PsiI; X, XmaI; S, SpeI. (D) Confirmation of the total LoxP collapse and generation of KO mice by PCR from genomic DNA using primers F1, R1, and R2 shown in (A).

A conditional PGC1βKO was generated using a triple LoxP targeting vector (Figure 1A) as detailed in the Material and Methods. Murine PGC-1β is alternatively spliced at the 5′ end [7] and at exon 4 [8,10]. Exons 4 and 5, which encode two of the nuclear hormone receptor interacting motifs (LXXLL), were deleted. Ablation of these exons also introduced a premature stop codon. PCR and Southern analyses of the selected clone confirmed the presence of the single LoxP in intron 5 and that the embyronic stem (ES) cell clone contained all three LoxP sites as designed (Figure 1B and 1C). Germ line transmission of the modified *PGC-1*β allele was confirmed by PCR analysis of the *agouti* pups obtained from chimeras bred to C57BL/6 wild-type (WT) mice. Heterozygous triple-LoxP–containing mice were then bred with ROSA26Cre mice to generate mice heterozygous for the PGC-1β deletion. Total LoxP site recombination was detected using PCR from genomic DNA (Figure 1D) and RT-PCR from isolated RNA. Intercross breeding of heterozygous germ-line knockout animals produced litters that did not deviate significantly from the expected Mendelian ratio of genotypes, and the size of litters from PGC1βKO heterozygous crosses was normal (averaging seven to eight pups per breeding event per female).

### Effect of PGC-1β Ablation on Energy Balance: Reduced Body Weight and WAT Content Consistent with Increased Energy Expenditure

**Figure 2:**
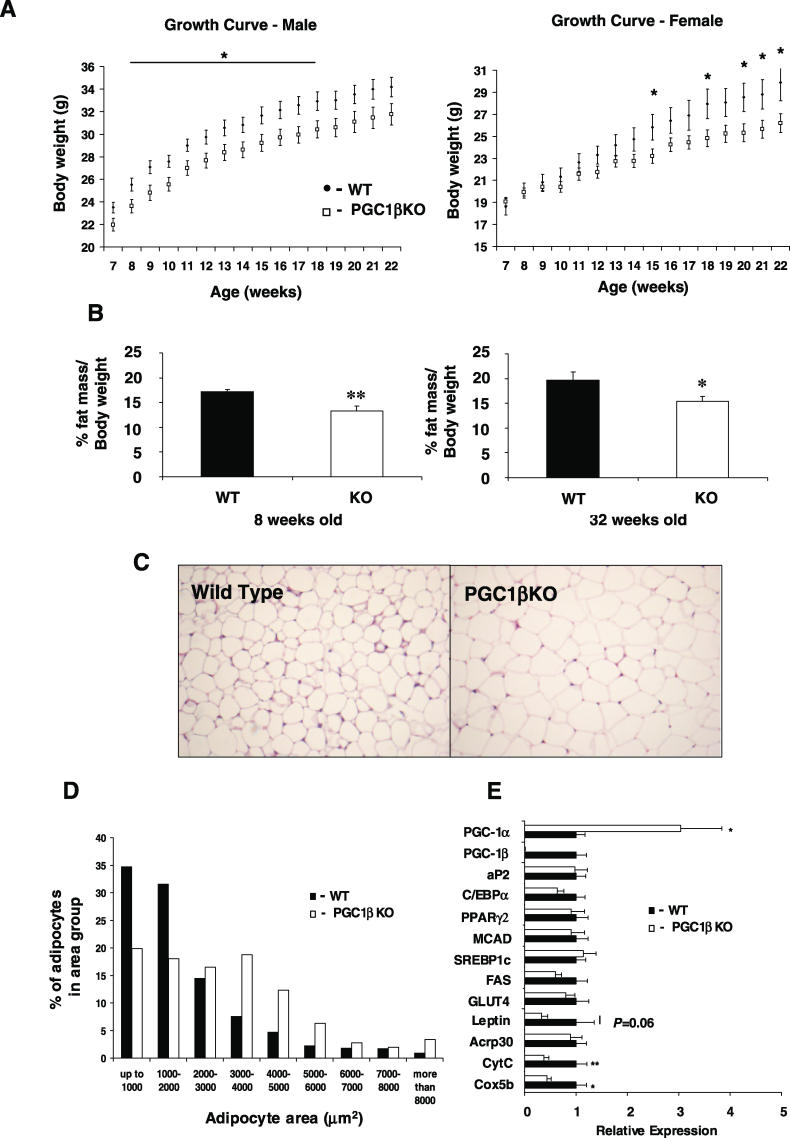
The PGC1βKO Mouse Has Altered Metabolism under Standard Environmental Conditions (A) Growth curves of male (left panel) and female (right panel) mice on normal diet. WT (solid circles) and PGC1βKO (open circles) mice, *n* = 18–21 mice per group. (B) Assessment of fat content by DEXA in 8- and 32-wk-old male WT (solid bars) and PGC1βKO mice (open bars), *n* = 8–12 mice per group. (C) Representative histological sections of tissues from WAT (*n* = 6). (D) Size distribution of adipocytes from WT and PGC1βKO mice. Two fields from each section from epididymal adipose tissue depot (*n* = 4 mice per genotype) were analysed to obtain the mean cell area per animal. (E) Epididymal WAT gene expression from 12-wk-old PGC1βKO (white bars) and WT littermates (black bars). Individual measurements are standardized using 18S, and then the average of the WT group was set to 1. *n* = 5–8 mice per group.

No differences in food intake, total energy intake, total energy output as faeces, and no change in the ratio of energy in (as food) to energy out (as faeces) (Table S1) were detected in 12-wk-old male mice selected for equal body weight. Oxymax analysis revealed increased minimum metabolic rate at 22 °C in 9-wk-old male PGC1βKO mice (Table 1). No differences in spontaneous activity (unpublished data) or in respiratory exchange ratio (RER) were detected, suggesting that there was no difference in whole-body substrate-type utilization. Consistent with an elevated minimum metabolic rate at ambient temperature, PGC1βKO mice weighed less than their WT littermates (Figure 2A). A similar pattern was also observed in female PGC1βKO mice.

**Table 1:**
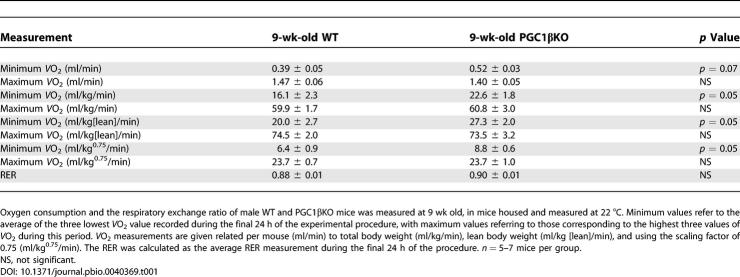
Assessment of Energy Expenditure on 9-wk-Old Male Chow-Fed PGC1βKO and WT Mice

Body composition analysis using dual energy x-ray absorptiometry (DEXA) confirmed decreased body weight at the expense of fat mass rather than lean mass in both young (8-wk) and older (27-wk) male PGC1βKO mice compared to WT littermates (Figure 2B). This was confirmed in an independent group of weight-matched 14-wk-old male PGC1βKO mice that had smaller gonadal adipose tissue depots than WT littermates. Other organs examined did not show differences in their weights relative to body weight (Table S2). Histological analysis of male gonadal WAT from PGC1βKO mice showed a reduced fraction of small adipocytes (up to an area of 2,000 μm^2^) and an elevated fraction of larger adipocytes in the PGC1βKO WAT (Figure 2C and 2D). This pattern for larger white adipocytes was also seen in male subcutaneous and omental WAT and in female gonadal WAT (Figure S1). Therefore, when on a chow diet, the PGC1βKO mice had less total WAT content than WT did, but it was composed of larger adipocytes. Histological analysis of other metabolically relevant tissues—including BAT, liver, skeletal muscle, and pancreas—did not reveal morphological alterations in PGC1βKO mice under basal conditions (Figure S2).

Gene expression analysis of gonadal WAT from male PGC1βKO mice and WT littermates showed no significant change in markers of adipogenesis including C/EBPα, PPARγ2, *aP2*, and *GLUT4* (Figure 2E). There was a significant up-regulation of PGC-1α expression in WAT in the absence of PGC-1β (Figure 2E). Previously, increased PGC-1α expression in WAT has been associated with the development of a BAT-like phenotype [21]. However, increased expression of PGC-1α was not sufficient to increase *UCP1* and D2-deiodinase expression in PGC1βKO WAT (unpublished data). Combined with the WAT morphology data, elevation of PGC-1α in the PGC1βKO WAT is not enough to result in clear BAT-like adipose tissue. However, ablation of PGC-1β resulted in reduced expression in the ETC genes *CytC* and *Cytochrome Oxidase* subunit *(Cox) 5b* and also in *leptin* (*p* = 0.06).

Ablation of PGC-1β did not result in differences in basal levels of plasma glucose or insulin. Similarly, glucose tolerance tests and insulin tolerance tests (GTTs and ITTs, respectively) performed on 16-wk male and female mice either on chow or after a 13-wk HFD did not reveal differences among genotypes (Figure S3). Therefore, the PGC1βKO mouse is leaner, has increased energy expenditure, and remains insulin sensitive.

### Reduced Expression of ETC Components Despite Increased PGC-1α mRNA Expression in the BAT of PGC1βKO mice

**Figure 3:**
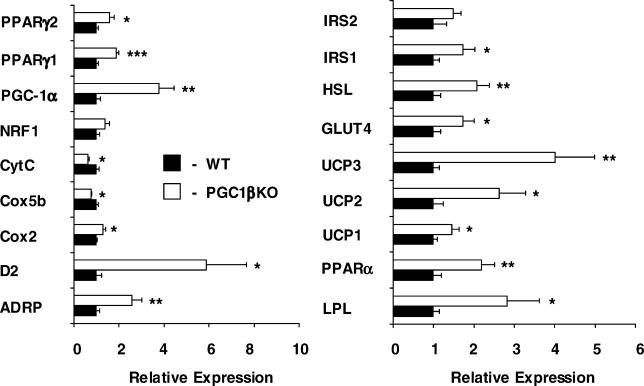
PGC-1β Ablation Results in Major Changes in BAT Metabolic Gene Expression Expression levels of mRNA were assessed on interscapular BAT from 12-wk-old male WT (black bars) and PGC1βKO (white bars) mice. Individual measurements are standardized using 18S, and the average of the WT group set to 1. *n* = 5–7 mice per group.

**Figure 4:**
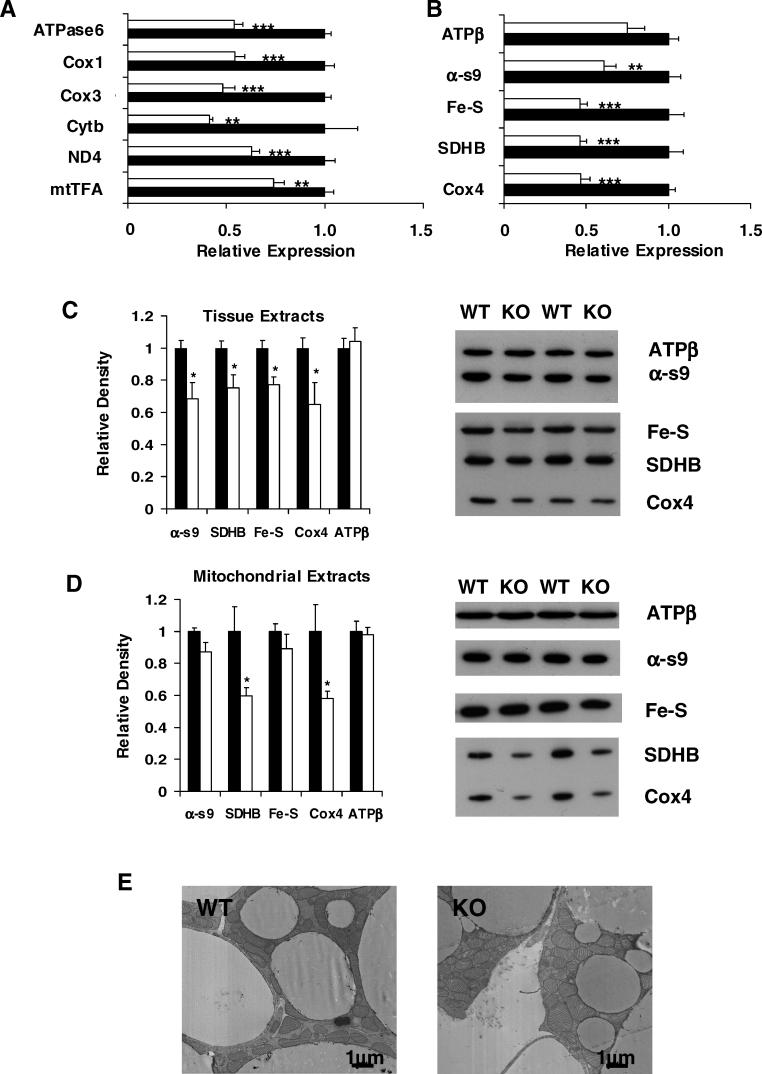
PGC-1β Ablation Reduces ETC Gene and Protein Expression but Mitochondrial Volume Fraction is Unaffected (A and B) Expression levels of (A) nuclear-encoded and (B) mitochondrially encoded genes were assessed on interscapular BAT from 12-wk-old male WT (black bars) and PGC1βKO (white bars) mice. Individual measurements are standardized using 18S, and the average of the WT group set to 1. *n* = 5–7 mice per group. (C and D) BAT protein levels of ETC and OxPhos components from 15-wk-old female mice were assessed by western blotting of samples from (C) tissues and (D) mitochondrial fractions. WT, black bars and PGC1βKO, white bars. Complex I, α-subcomplex 9 (α-s9); complex II, succinate dehydrogenase subunit B (SDHB); complex III, Fe-S core protein (Fe-S); complex IV, Cox4; complex V, ATP synthase subunit β (ATPβ). *n* = 5–6 mice for each protein, with the average value of the WT group set to 1. Representative blots showing two samples from each genotype. (E) Representative electron micrographs from BAT of WT (left panel) and PGC1βKO (right panel) mice.

Examination of BAT in chow-fed 12-wk-old PGC1βKO male mice and WT littermates showed major changes in the expression of genes involved in intermediary metabolism (Figure 3). At ambient temperature, we identified a 4-fold compensatory increase in the expression of PGC-1α when PGC-1β was ablated. We also observed a 5.9-fold elevation in the thyroid-dependent pro-thermogenic enzyme *D2-deiodinase*. These changes were associated with up-regulation in PGC1βKO mice of genes involved in glucose and fatty acid metabolism (including *GLUT4, PPARα, ADRP, LPL,* and *HSL*) as well as elevations in PPARγ1 and 2 and all three UCP isoforms. These changes suggested that increased PGC-1α expression was overcompensating for a lack of PGC-1β. Despite this up-regulation of PGC-1α, ablation of PGC-1β still resulted in a specific decreased expression of genes involved in mitochondrial ETC such as *Cox5b* and *CytC*. In addition, a further range of mitochondrially encoded (Figure 4A) and nuclear-encoded (Figure 4B) ETC mRNAs were also reduced in PGC1βKO mouse BAT. The mitochondrially encoded *Cox2* was the only ETC subunit measured in PGC1βKO BAT with increased expression. Western blotting analysis of ETC components in BAT lysates from 15-wk-old female mice revealed decreases in subunits from complexes I to IV in PGC1βKO mice (Figure 4C). Similarly, assessment of succinate dehydrogenase subunit B (SDHB, complex II) and Cox4 protein levels in isolated mitochondrial preparations were both reduced in PGC1βKO mice (Figure 4D). ATP synthase subunit β was unchanged. Taken together, these results demonstrate defective ETC expression and protein composition in the BAT of PGC1βKO mice, which cannot be fully compensated for by up-regulation of PGC-1α. Despite these defective changes, mitochondrial volume fraction was only marginally affected in BAT of PGC1βKO mice (Figure 4E, WT versus PGC1βKO: 5.0 ± 0.3% versus 4.4 ± 0.2%, *n* = 4 mice per group, *p* = not significant [NS]) and there was no difference in BAT lipid content as determined by light microscopy (WT versus PGC1βKO: 13.7 ± 0.8 versus 13.1 ± 0.9, *n* = 4 mice per group, *p* = NS). This suggests that the up-regulation of PGC-1α in BAT can compensate some, but not all, aspects of the mitochondrial system in this tissue.

### PGC1βKO Mice Are Cold-Tolerant but Display Abnormal Responses to Norepinephrine-Induced Energy Expenditure

**Figure 5:**
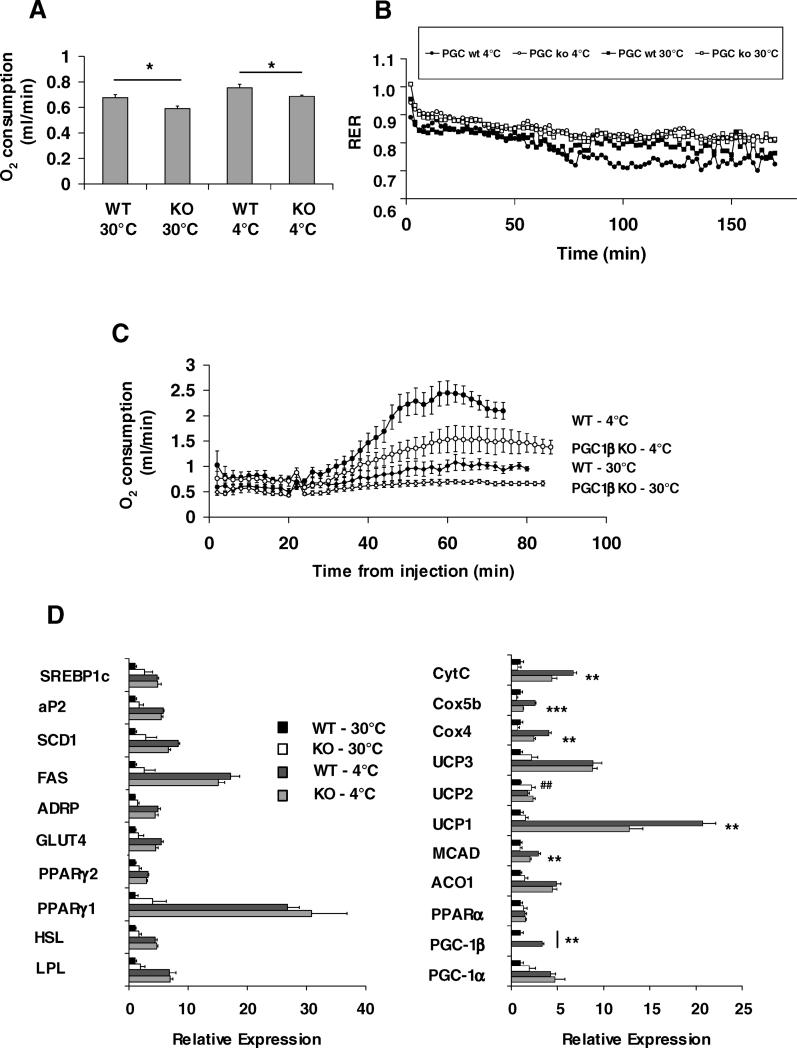
Thermoneutral and Cold-Adapted PGC1βKO Mice Display Reduced Resting and NE-Stimulated Energy Expenditure Male WT and PGC1βKO mice were acclimatised to 30 °C or 4 °C as stated in the Materials and Methods. (A) Oxygen consumption per mouse (ml/min). Similar results were obtained when the data were standardised using the body weight (kg)^0.75^ scaling; WT, black bars; PGC1βKO, white bars. (B) RER. Solid circles, WT at 4 °C; open circles; PGC1βKO at 4 °C; solid squares, WT at 30 °C; open squares, PGC1βKO at 30 °C. (C) Oxygen consumption per mouse (ml/min) measured post-administration of 1 mg/kg norepinephrine. (D) BAT mRNA expression in thermoneutral and cold-adapted mice. WT thermoneutral, black bars; PGC1βKO thermoneutral, white bars; WT cold-adapted, dark gray bars; PGC1βKO cold-adapted, light gray bars. Individual measurements are standardized using 36B4 and the average of the WT, thermoneutral group was set to 1. *n* = 4–5 mice per genotype per condition for each experiment. * indicate a significant difference between cold-adapted WT and PGC1βKO mice. ## indicates a significant difference between thermoneutral WT and PGC1βKO mice.

A stepwise acclimatisation to cold exposure (4 °C) and towards thermoneutrality (30 °C) was performed on male mice that were 16 wk old at the end of the 3-wk cold-acclimatisation process. The rationale for these experiments was to identify the contribution of PGC-1β to energy expenditure in these two directly opposing thermogenic scenarios. PGC1βKO mice survived at 4 °C after this acclimatisation process. BAT pad weight was similarly increased by cold-acclimatisation in both WT (BAT weight in mg for 30 °C versus 4 °C: WT 117.8 ± 7.1 versus 145.5 ± 9.4, *p* < 0.05, *n* = 5 mice per group) and PGC1βKO mice (107.9 ± 9.1 versus 160.0 ± 10.3, *p* < 0.01, *n* = 4 mice per group), without differences in BAT weight between the genotypes in either condition. Cold-acclimatised PGC1βKO mice had decreased basal metabolic rate compared to WT (Figure 5A) and had a higher RER than WT mice, suggesting that PGC1β-deficient adaptation to cold exposure may involve preferential use of carbohydrates (Figure 5B). Thermoneutrality studies showed that contrary to the increased resting metabolic rate observed at ambient temperature, when PGC1βKO mice are housed and measured at thermoneutrality, they have reduced basal metabolic rate compared to WT (Figure 5A). Of note, PGC-1α up-regulation observed in PGC1βKO mice at ambient temperature was prevented at thermoneutrality (see below).

To test the maximal BAT-based nonshivering thermogenesis capacity, we used a norepinephrine (NE) challenge (Figure 5C). Whereas NE provoked the expected large (almost 4-fold) increase in O_2_ consumption in cold-adapted WT mice, the same stimulus in cold-adapted PGC1βKO mice only reached 60% of the WT response. (Figure 5C). NE administration to mice kept at thermoneutrality revealed that whereas WT mice exhibited a physiological small increase in O_2_ consumption after being given NE, O_2_ consumption in the PGC1βKO mice housed at thermoneutrality was essentially unaffected by NE. This demonstrates that the ability of the BAT to mediate thermoregulatory thermogenesis after a period of cold acclimatisation or during thermoneutrality is blunted by the absence of PGC-1β.

Gene expression of BAT from cold-acclimatised and thermoneutrally maintained mice was analysed. Comparison of gene expression patterns between WT and PGC1βKO mice housed at 30 °C (Figure 5D) revealed the same differences between the genotypes as previously seen in mice housed at ambient temperatures (Figure 3). In addition, many of the genes examined concerning fuel handling (e.g., *SREBP1c, GLUT4, LPL, PPARγ*) were similar in cold-acclimatised WT and PGC1βKO mice (Figure 5D, left panel). However, cold-acclimatised PGC1βKO BAT still had decreased expression of the ETC genes *Cox4, Cox5b,* and *CytC* (Figure 5D, right panel). Similarly, there was significantly lower expression of UCP1 and MCAD in cold-acclimatised PGC1βKO BAT than in WT. With cold exposure, PGC-1α mRNA was increased to a similar extent in both WT and PGC1βKO mice, and PGC-1β expression was increased in WT mice. Also, as indicated above, there was no difference in PGC-1α expression between the genotypes at thermoneutrality. As expected, cold acclimatisation increased levels of UCP1 protein in WT (total interscapular BAT UCP1 protein 30 °C versus 4 °C, standardised to WT 4 °C: 12.9 ± 2.8 versus 100.0 ± 7.4; *n* = 5 mice per group) and also in PGC1βKO mice (14.9 ± 2.9 versus 140.9 ± 19.9; *n* = 4 mice per group). Total BAT UCP1 protein was near-significantly elevated (*p* = 0.07) in cold-acclimatised PGC1βKO mice compared to cold-acclimatised WT mice. Thus, the inhibition of cold-acclimatisated BAT activity in PGC1βKO mice occurs as a result of reduced ETC expression, despite otherwise normal BAT recruitment and UCP1 protein expression.

### Gene Expression Pathway Analysis in PGC1βKO Tissues Shows Global Impairment in Mitochondrial Function and Substrate Use

Binomial pathway enrichment analysis using pathways from the Kyoto Encyclopedia Genes and Genomes (KEGG) was performed on gene expression from spotted arrays generated from BAT of PGC1βKO and WT mice to identify alterations in whole genetic pathways (Table 2). Down-regulated KEGG pathways include OxPhos, ubiquinone biosynthesis, and ATP synthesis. In addition, we saw decreases in glycolysis/gluconeogenesis, the citric acid cycle, and many of the amino acid metabolism pathways that feed substrates into these systems. Only purine metabolism and glycerolipid metabolism pathways were significantly up-regulated in BAT. A full list of altered KEGG pathways can be found in Dataset S1. Similar gene expression pathway analysis was also performed in liver and heart tissues. We identified only two KEGG pathways, OxPhos, and ATP synthesis that were decreased in all three tissues—BAT, heart, and liver (Table S3). This suggests a global role for PGC-1β in the maintenance of normal gene expression encoding for mitochondrial proteins.

**Table 2:**
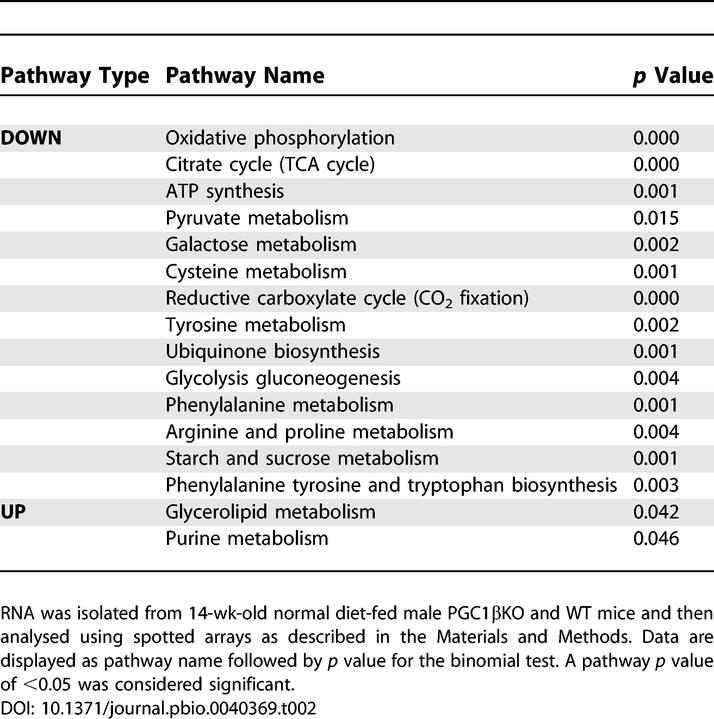
Major KEGG Pathways Are Changed in PGC1βKO Mouse BAT

### Tissue Mitochondrial Activity, but Not Isolated Mitochondrial Function, Is Impaired in PGC1βKO Soleus Muscle

**Figure 6:**
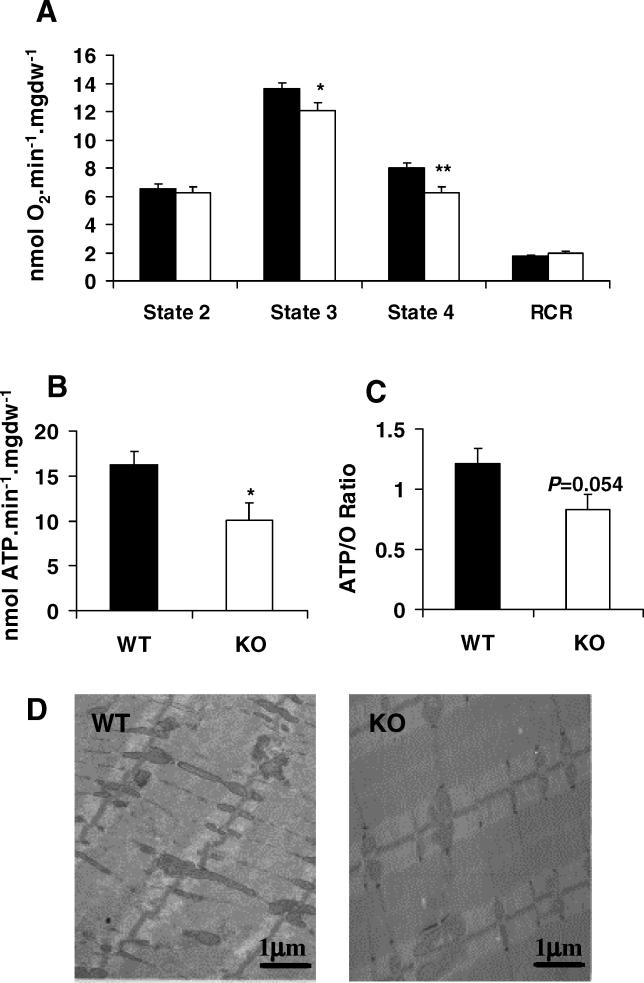
Isolated Soleus Fibres from PGC1βKO Mice Have Reduced Mitochondrial Activity Soleus fibres were isolated from WT (black bars) and PGC1βKO mice (white bars) and permeabilised to allow measurement of tissue-associated mitochondrial function. (A) Mitochondrial respiratory parameters for state 2 (*V*_0_), state 3 (*V*_ADP_), state 4 (*V*_oligomycin_), and respiratory control ratio (RC). (B) ATP synthesis rates in permeabilised soleus fibres. (C) ATP/O ratio in permeabilised soleus fibres. Data are standardised to mg of muscle dry weight (mgdw). *n* = 9 WT mice, 11 PGC1βKO mice. (D) A representative electron micrograph of soleus muscle from WT (left panel) and PGC1βKO (right panel) mice.

We assessed whether reduced expression of ETC genes were associated with functional defects in permeabilised soleus muscle fibres (Figure 6). Both state 3 and state 4 mitochondrial respiration were reduced in PGC1βKO fibres, suggesting reduced electron chain capacity and OxPhos activity (Figure 6A). There was no change in the respiratory control ratio (RCR; state 3/state 4), suggesting that the differences between WT and PGC1βKO mice were not related to intrinsic differences in mitochondria function but to reduced numbers of mitochondria per fibre. In agreement with this, ATP synthesis was decreased in PGC1βKO fibres (Figure 6B), and a trend for decreased ATP/O ratio in PGC1βKO muscle fibres was also observed (Figure 6C). This suggested that PGC1βKO soleus fibres not only produce less ATP than WT fibres did, but they were also less efficient at converting reduced substrate into ATP. Gene expression analysis of 12-wk-old male soleus muscles demonstrated that PGC1βKO mice have reduced ETC expression *(Cox4* and *Cox5b),* which corresponds to reduced soleus mitochondrial capacity (Figure S4). These results were supported by a decreased mitochondrial volume fraction in the soleus of PGC1βKO mice performed by stereological analysis (WT versus PGC1βKO: 20.4 ± 0.7% versus 16.8 ± 0.8%, *p* = 0.012) (Figure 6D).

To assess whether this oxidative defect could be attributed to the functionality of individual mitochondria, we measured oxidative activity of isolated soleus mitochondria. In all states examined, there was no significant difference between WT and PGC1βKO-derived soleus mitochondria (Figure S5). In addition, no differences amongst genotypes were observed when RCR (Figure S5B) or proton leak were measured (Figure S5C).

### PGC-1β Ablation Reduces Mitochondrial Content of the Heart and Blunts the Effect of Adrenergic Stimulation on Heart Rate

**Figure 7:**
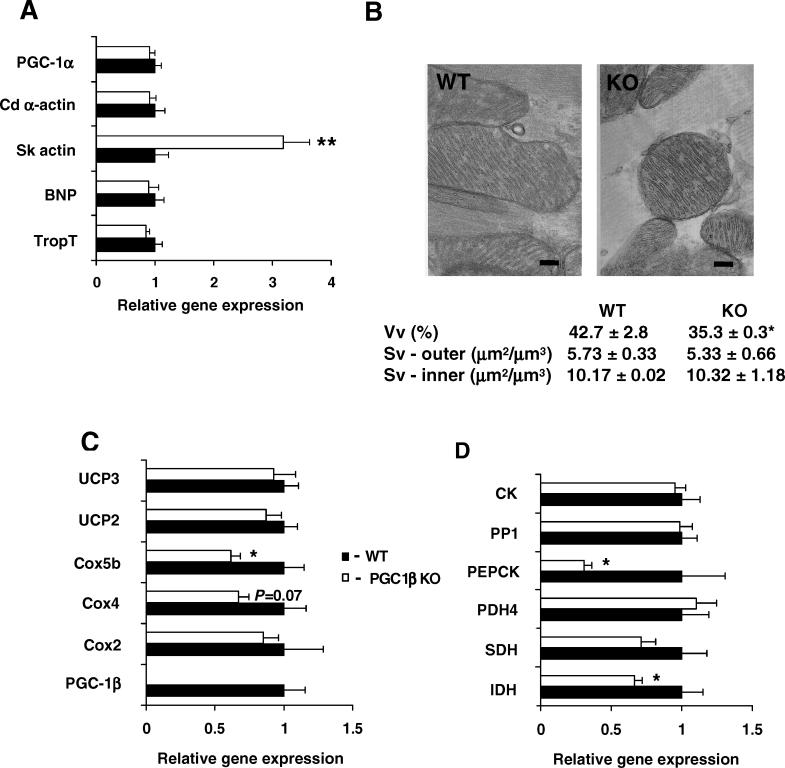
Alterations in Gene Expression and Mitochondrial Dimensions in Hearts of PGC1βKO Mice (A) Expression levels of mRNA were assessed on hearts from 24-wk-old male WT (black bars) and PGC1βKO (white bars) mice. Individual measurements are standardized using 18S, and the average of the WT group set to 1. *n* = 5–7 mice per group. (B) A representative electron micrograph of mitochondria from WT (left panel) and PGC1βKO (right panel) hearts. The bar indicates a measurement of 200 nm. (C) mRNA expression of key genes for mitochondrial function in 24-wk-old WT and PGC1βKO mouse hearts. (D) mRNA expression of key genes for metabolic function in 24-wk-old male WT and PGC1βKO mouse hearts.

We hypothesized that defective PGC-1β may result in electromechanical myocardial dysfunction in the hearts of PGC1βKO mouse. Heart weights from PGC1βKO mice were similar to WT in chow-fed 14-wk-old male mice (Table S2). In addition, under basal conditions, PGC1βKO hearts showed no histological abnormalities compatible with fibrosis or inflammatory infiltrates (unpublished data). The expression of molecular markers of cardiac hypertrophy, such as *α-actin, natriuretic peptide precursor type B,* or *troponin I,* were all normal in 24-wk-old male PGC1βKO hearts (Figure 7A). The only transcriptional change that can be associated with myocardial dysfunction was the up-regulation of *skeletal muscle actin* in PGC1βKO compared with WT mice. PGC-1α mRNA expression was also unchanged in PGC1βKO hearts. PGC1βKO hearts had a decreased mitochondrial volume fraction as indicated by stereological analysis (42.8 ± 2.8 % versus 35.3 ± 0.3 %, *p* = 0.05: Figure 7B). Despite this result, PGC1βKO and WT heart mitochondria were similarly packed between myofibres, and there were no changes in cristae surface density, *S*_v_ (per unit volume of mitochondria). This suggests that the internal structure of the mitochondria was normal in the PGC1βKO hearts. In agreement with the gene expression pathway analysis, the expression of *Cox4* (*p* = 0.07) and *Cox5b* was down-regulated in heart of 14-wk-old PGC1βKO compared with WT mice, whereas *Cox2* expression was unchanged (Figure 7C).

**Figure 8:**
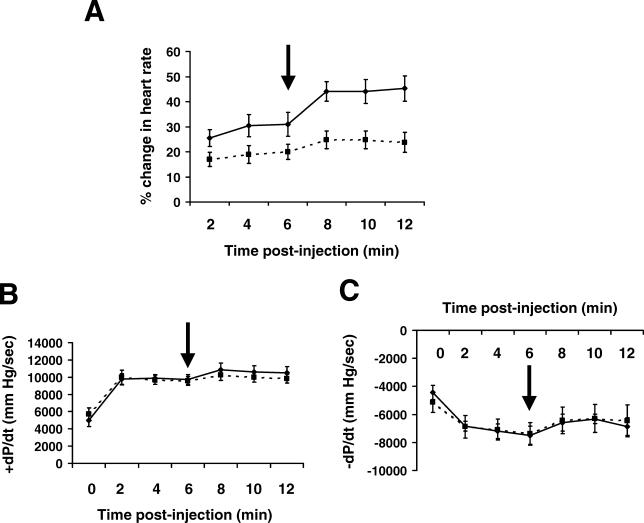
PGC1βKO Hearts Display a Blunted Heart Rate Response to Dobutamine Stimulation In Vivo PGC1βKO and WT littermates (male, 26-wk-old) were treated as stated in Material and Methods and infused with 10 or 40 ng/min/g BW dobutamine to measure in vivo hemodynamic responses WT, solid line; KO, dashed line. (A) Percentage change in heart rate from basal during dobutamine infusion. (B and C) Measurement of ventricular performance, *dP*/*dt*, during infusion. The arrow marks the increase in dobutamine concentration in the infusion from 10 to 40 ng/min/g BW. *n* = 5 mice per genotype.

Male, 26-wk-old PGC1βKO and WT littermates were challenged with acute infusion of the β_1_,α_1_-adrenergic selective agonist dobutamine. Haemodynamic recordings showed that baseline heart rate and left ventricular contractility (+*dP*/*dt* or –*dP*/*dt*) were similar between genotypes. As expected during infusion of 10 ng dobutamine per min per g body weight (ng/min/g BW), WT mice increased their heart rate, an effect that was blunted in PGC1βKO mice (Figure 8A). This inability to increase heart rate was even more evident using a 40 ng/min/g BW dobutamine infusion (*p*< 0.05 for 8, 10, and 12 min post-injections). Ventricular performance during the dobutamine challenge was the same in WT and PGC1βKO mice (Figure 8B and 8C).

### PGC-1β Modulates the Response of the Liver to an Acute HFD Challenge

**Figure 9:**
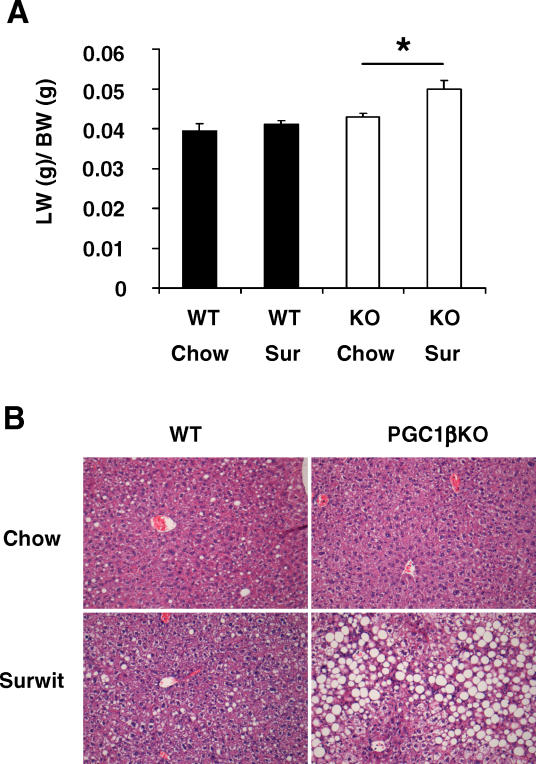
PGC1βKO Mice Demonstrate Increased Liver Mass and Development of Fatty Liver after 24 h HFD Female 8-wk-old mice were given normal chow or Surwit HFD (Sur) for 24 h. Tissues were then collected for analysis. (A) Liver weight, as standardized by body weight (LW/BW) for WT (black bars) and PGC1βKO (white bars) after 24 h diets. (B) Representative histological sections from mice given normal or Surwit diet for 24 h. *n* = 6–7 mice per group.

Gene expression analysis of fed-state 12-wk-old male mouse livers showed ablation of PGC-1β but did not result in major changes in mRNA expression levels (Figure S6). Only *Cox4* was decreased in the PGC1βKO livers, a result that was in agreement with gene expression pathway analysis (Table S3). When 8-wk-old female PGC1βKO mice were challenged with a 24-h period of Surwit HFD, liver mass increased as a proportion of body weight (Figure 9A). This effect was not observed in WT mice fed the same diet. Histological analysis revealed that PGC1βKO mice fed the Surwit diet developed severe hepatic steatosis compared to WT mice (Figure 9B). Increased fat deposition in PGC1βKO livers was associated with normal plasma non-esterified fatty acids (NEFA) levels (Table 3). As expected, plasma triglyceride levels increased in WT mice in response to 24-h HFD, however plasma triglyceride levels were significantly lower in PGC1βKO than WT mice when fed the Surwit diet. When compared to WT mice, the PGC1βKO mice had lower basal levels of high-density lipoprotein (HDL) and LDL-associated cholesterol and elevated VLDL-associated cholesterol. Administration of a fat-enriched diet increased the amount of cholesterol associated with VLDL, HDL, and LDL particles in WT mice (Table 3). However, PGC1βKO mice had inappropriately low levels of VLDL and blunted increases on total cholesterol and HDL- and LDL-associated cholesterol in plasma. Thus, defective PGC-1β results in lipid accumulation in liver and decreased circulating triglyceride-rich VLDL lipoproteins following a high-dietary lipid load.

**Table 3:**
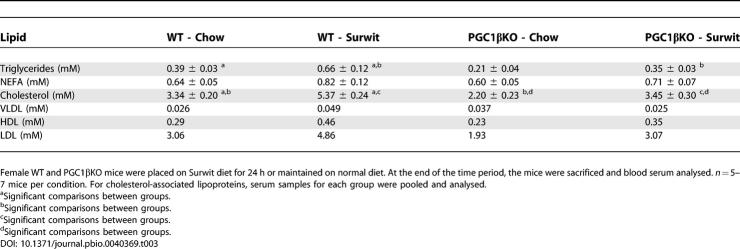
Serum Lipid Biochemistry from 24-h Surwit Diet–Fed WT and PGC1βKO Mice

Of interest and contrary to previous reports, we were unable to detect increases in PGC-1α and PGC-1β mRNAs in response to acute HFD in WT liver (Figure S7). We observed that *SCD-1* and *HMG-CoA reductase* were significantly elevated in WT livers in response to Surwit diet compared to chow WT controls and that similar changes were not observed in PGC1βKO mouse livers after the Surwit diet (Figure S7).

## Discussion

Accumulating evidence indicated that PGC-1β may play a role in energy homeostasis through its effects on substrate metabolism and mitochondrial activity [4,6,7,13–15,19]. However, the function and relevance of PGC-1β in the control of whole-organism energy metabolism is not well defined. To address this question, we generated and phenotyped a PGC1βKO mouse model. The PGC1βKO mouse is viable and apparently healthy. However, using a phenotyping strategy that combines physiological stress challenges guided by information obtained from a systems biology approach, we found that PGC1βKO mice have a general defect in mitochondrial function, a defect that is partly compensated for by mechanisms that are usually involved in adaptation to increased energy demands, such as up-regulation of PGC-1α.

The effect of PGC-1β controlling mitochondrial activity was investigated at the mRNA level using spotted array technology and bioinformatics pathway analysis. This approach showed that deletion of PGC-1β results in a significant mitochondrial phenotype. Multi-tissue comparisons demonstrated that PGC-1β controls the level of expression of ETC and OxPhos genes across all the organs studied and that this defect can only be partially compensated for in BAT and WAT by up-regulation of PGC-1α. However, the decrease in mRNA expression of ETC components is not necessarily seen at the protein level. These data suggest that although PGC-1β may be a controller of mitochondrial gene expression, in the absence of PGC-1β, additional factors such as protein degradation may be counter-regulated in specific tissues in an attempt to normalise levels of mitochondrial activity.

Our initial hypothesis was that dysregulation of ETC gene expression would increase the likelihood that the PGC1βKO mouse would become obese. However, despite its mitochondrial phenotype, the PGC1βKO mouse showed elevated resting metabolic rate and lower body weight compared to wild type littermates under ambient room temperature conditions. This phenotype is possibly due to the compensatory increase in expression of PGC-1α and its target genes in BAT. The increase in BAT PGC-1α expression may cause the higher degree of energy expenditure and relatively conserved mitochondria volume fraction despite reductions in ETC gene and protein expression. PGC-1α was also up-regulated in PGC-1β–deficient WAT, but its induction was not robust enough to produce a BAT-like histology or induction of typical BAT genes. Instead, within the reduced WAT content of the PGC1βKO mice, there was a greater proportion of larger, hypertrophic adipocytes. This result suggests that PGC-1β may play an as yet unknown role in white adipocyte biology, and the nature of this role requires further investigation.

Of interest, despite marked elevation of PGC-1α levels in PGC1βKO BAT, we did not observe a full restoration of the expression of mitochondrial genes back to WT levels. However, unlike the heart or skeletal muscle, organs where PGC-1α was not up-regulated, the mitochondrial fraction of BAT tissue was preserved in PGC1βKO mice. Thus, there may be partial functional overlap between PGC-1α and PGC-1β, concerning mitochondrial structure and function, which allows the development of an appropriate mitochondrial BAT content but fails to correct deficiencies in ETC composition. BAT was the only tissue out of three metabolically relevant tissues examined using electron microscopy that displayed relatively normal mitochondrial fraction, suggesting that further tissue function–specific mechanisms may be able to correct for PGC-1β ablation. However, the BAT gene expression pattern in PGC1βKO mice also showed altered expression of genes involved in intermediary metabolism, a pattern of gene expression that may also be primarily the result of PGC-1α up-regulation. Our conclusion from these gene expression analyses is that whereas PGC-1β may be required to set the basal level of mitochondrial ETC gene expression, it is not essential for normal expression of metabolic pathways such as glycolysis, the citric acid cycle, and fatty acid oxidation.

We hypothesized that the elevated resting metabolic rate seen in ambient-temperature housed PGC1βKO mice may be reversed under conditions of thermoneutrality, particularly if it was caused by compensatory up-regulation of PGC-1α. Under thermoneutral conditions, levels of PGC-1α gene expression are suppressed in BAT in response to the lower thermogenic and therefore oxidative demands of life at 30 °C. At thermoneutral conditions, PGC-1α mRNA expression was equivalent in WT and PGC1βKO BAT. Interestingly, under thermoneutral conditions, the PGC1βKO mouse exhibited reduced basal metabolic rate compared to the WT mouse. The energy expenditure measurements with mice acclimatised to 4 °C and 30 °C were performed at 33 °C. This measurement at thermoneutrality gives an indication of the basal metabolic rate. Under these experimental conditions, we can conclude that the PGC1βKO mouse has a reduced basal metabolic rate. The data from mice housed in ambient (22 °C) conditions were also generated at ambient conditions (Table 1). Therefore, this measurement represents energy expenditure in “normal” environmental conditions, when the body weights of the PGC1βKO mice are lower than WT. Under these experimental conditions, this measurement is composed of both basal metabolic rate and any additional metabolic effort required to maintain body temperature in this suboptimal thermal environment. Given that basal metabolic rate is reduced in 30 °C– and 4 °C–acclimatised PGC1βKO mice, it could be assumed that at ambient temperatures, basal metabolic rate is also reduced, but that the metabolic adaptation required for body temperature defense is actually greater in the PGC1βKO mouse. This suggests that PGC-1β may contribute to BAT thermogenesis in states of low metabolic demand (as is the case for BAT at 30 °C), whereas induction of PGC-1α regulates BAT thermogenesis as environmental temperatures fall.

It has been suggested that PGC-1β plays a role in BAT accumulation and function during cold exposure [6]. PGC1βKO mice tolerated cold acclimatisation, despite the fact that under these conditions, PGC1βKO mice had decreased ETC gene expression and had decreased maximal thermogenic capacity in BAT, as shown by the lower oxygen consumption rate in response to adrenergic stimulation. Both WT and PGC1βKO mice up-regulated PGC-1α to a similar extent under conditions of cold exposure, suggesting that there is a maximal capacity of PGC-1α up-regulation in BAT. Alternatively, survival in cold may also be facilitated by the ability of the mice to maintain some fraction of increased metabolic activity through continued shivering [22–24]. Therefore, although the total metabolic capacity provided by PGC-1α in cold-exposed PGC1βKO mice was sufficient for their survival, our results indicate that PGC-1β regulates a large fraction of the thermogenic capacity of the BAT tissue.

Studies focused in other metabolically relevant organs where PGC-1β is well expressed also indicated that ablation of PGC-1β resulted in impaired metabolic capacity. For example, mitochondrial volume fraction in soleus of PGC1βKO mice was decreased, and permeabilised soleus muscle fibres from PGC1βKO mice had reduced oxygen consumption and ATP synthesis compared to WT-derived fibres. Similar experiments using isolated soleus mitochondria from PGC1βKO mice failed to show any abnormalities in metabolic performance, indicating that metabolic defects in PGC1βKO soleus tissue relate to lower mitochondrial density relative to WT, although individual mitochondria retain similar metabolic properties.

The heart is the third oxidative tissue that is a major site of PGC-1β expression. Absence of PGC-1β in heart also led to a reduction of mitochondrial fraction, consistent with reduced cardiac expression of ETC genes. However, despite these potential bioenergetic defects, PGC1βKO mice have normal basal heart rates and ventricular contractility. Left ventricular function was also maintained after dobutamine treatment, suggesting that despite their mitochondrial defect, the PGC-1β–deficient hearts are able to adapt normally to imposed hemodynamic loads. The only difference in adrenergic stress that we observed between the PGC1βKO mice and WT was a blunting of the expected increase in heart rate in the PGC1βKO mice. A similar defect in heart rate regulation was also found in the PGC-1α knockout mice [14], suggesting that both PGC-1α and PGC-1β may have a direct effect in controlling the activity of heart pacemakers. Overall, our results indicate that ablation of PGC-1β impairs heart mitochondrial function but that this defect is not severe enough to induce heart failure. However, a more chronic intervention study would be required to establish the relative importance of these PGC isoforms in cardiac energy supply and control.

To this point, we have discussed exclusively a role for PGC-1β in oxidative metabolism. Nonetheless, PGC-1β may also play an important role in regulating hepatic lipid production [11,17]. Contrary to previous reports, we did not observe either PGC-1β induction after 24-h HFD in the liver of our control mice or differences in *FAS* and *SREBP1c* gene expression between controls and PGC1βKO mice. However, we did observe a reduction in circulating total cholesterol and alterations in the lipoprotein-associated cholesterol pattern in chow-fed conditions. Feeding for 24 h with a saturated fat–enriched HFD was associated with severe hepatic lipid accumulation and decreased total triglycerides, cholesterol, and VLDL and LDL-cholesterol plasma levels in PGC1βKO mice compared to WT. These results agree with a recent report suggesting that PGC-1β is a likely mediator of VLDL secretion by acting via interactions with Foxa2 to alter microsomal transfer protein expression [17]. However, it is also possible that PGC-1β is necessary for additional aspects of hepatic lipid handling, including modification of lipid storage pathways and for controlling the balance between fatty acid synthesis and oxidation. Indeed, our pathway analysis suggests that the ETC in liver is expressed at a lower level and this may contribute to the steatosis after acute HFD treatment.

Our results indicate that PGC-1β has a well-defined role in controlling mitochondrial gene expression and function in many different organs. Despite this, PGC-1β can be ablated without overt metabolic failure, at least in unstressed conditions. This may be due in part to robust compensatory mechanisms, as demonstrated by the up-regulation of PGC-1α expression in WAT and BAT. Interestingly, other organs such as liver, muscle, or heart did not show up-regulation of PGC-1α under the conditions investigated. Following from these differences in PGC-1α expression, it is likely that the lean phenotype of this mouse model at ambient temperature should be considered the result of overcompensation mediated by up-regulation of PGC-1α, at least in BAT and WAT. Conversely, defects observed in skeletal muscle, heart, and liver are more likely to be the result of the absence of PGC-1β given the lack of PGC-1α induction in those tissues. When considering the roles played by PGC-1α and PGC-1β, our results show that there are specific effects of PGC-1β that cannot be compensated for by PGC-1α. Taken altogether, our results indicate that PGC-1β seems to cover basal bioenergetic needs whereas PGC-1α provides the extra bioenergetic support required under conditions of increased energy demand.

## Materials and Methods

### Materials and reagents.

All reagents used in this paper were supplied from Sigma-Aldrich (St. Louis, Missouri, United States), unless stated.

### Animal care.

Animals were housed in a temperature-controlled room with a 12-h light/dark cycle. Food and water were available ad libitum unless noted. All animal protocols used in this study were approved by the UK Home Office, The Institutional Animal Care and Use Committee of the University of Utah, United States, and the Animal Ethics Committees of Gothenburg and North Stockholm, Sweden. Mice were cared for according to the Guiding Principles for Research Involving Animals and Human Beings.

### Generation of PGC1βKO mice.

A triple LoxP strategy was used to target the PGC-1β locus in order to generate mice with both standard and conditional KO alleles at this locus. The targeting vector was a ~8 kilobase (kb) 129/SvJ mouse genomic subclone containing a floxed neomycin phosphotransferase selectable marker cassette inserted into intron 3 and a single LoxP site inserted into intron 5 (Figure 1A). The targeting construct was electroporated into R1 ES cells, and neomycin-resistant clones were selected in G-418-containing media. The clone was injected into C57Bl/6 blastocysts and chimeric males were crossed to C57Bl/6 females. Heterozygous triple LoxP mice were then bred to ROSA26Cre [25] mice in order to generate heterozygous PGC-1β KO mice which had undergone Cre-mediated deletion of the intervening region of DNA between the outermost LoxP sites. PCR analysis gave a product of approximately 0.5 kb for the Cre-recombined allele and products of approximately 2.8 and 0.6 kb with the WT allele (Figure 1D). Heterozygous PGC-1β KO mice were then intercrossed to generate mice that were homozygous for the PGC-1β deletion. Null mutants were verified using RNA prepared from heart and skeletal muscle to demonstrate that exons 4 and 5 had been deleted in the PGC1βKO mice. Mice used for this research were backcrossed between three and six times to a C57BL6/J background. Littermate controls were used for all experiments. A more complete description for this section can be found in the Protocol S1.

### Long-term feeding and growth studies.

Mice were placed at weaning (3-wk-old) on normal chow diet (12% fat, 62% carbohydrates, and 26% protein with a total energy content of 12.6 kJ/g) (R3 diet, Lactamin AB, Stockholm, Sweden). Mouse weights were taken at the same time each week, until the end of the specific protocol period. Mice were routinely housed at 22 °C, except for those used for the BAT activity and cold acclimatisation experiments (see below).

### Acute dietary measurements and interventions.

To examine 48-h food intake, cages (23 × 16 cm) were prepared with normal chow and incubated at 80 °C for 1 h to correct for any differences in humidity. After 2 h at room temperature, the cages were accurately weighed. 12-h-fasted mice were put in preweighed cages with free access to food and water. After 48 h, the mice were removed and all faecal matter was collected. The cages were reincubated at 80 °C in order to dry out waterspill and urine, and then reweighed after 2 h cooling. The difference in weights of the cage before and after the 48-h assessment produced the weight of food consumed. For measurement of energy content of faeces and food, samples were dried at 55 °C overnight and stored in airtight containers at −20 °C until assayed. The gross energy content of the dried samples was determined using a bomb calorimeter (C5000, IKA Werke GmbH & Co., KG, Germany). To assess the effect of 24-h high-fat feeding, 8-wk-old female mice were assigned into two groups: ad libitum normal food or ad libitum Surwit diet (58% of calories derived from fat, predominantly hydrogenated coconut oil; D12331, Research Diets, New Brunswick, New Jersey, United States). Start of the 24-h period was 9 am. At the end of the time period, mice were killed and dissected as above. Water was freely available during all procedures.

### Body composition and indirect calorimetry.

For body composition analysis, dual energy x-ray absorptiometry (DEXA, GE Medical Systems Lunar Corporation, Madison, Wisconsin, United States) was performed on isoflurane anaesthetized mice as previously described [26]. Oxygen consumption (VO_2_) and carbon dioxide production (VCO_2_) were measured using the Oxymax system (Columbus Instruments International, Columbus, Ohio, United States) [27]. Further details can be found in Protocol S1.

GTTs and ITTs on mice fed chow and HFDs were performed as previously described [26].

### Cold acclimatisation and BAT activity.

PGC1βKO mice and WT littermates were exposed to 30 °C for 3 wk, placed at 18 °C for 1 wk, and then exposed to 4 °C for 3 wk or kept at 30 °C for the duration of the protocol. For both 30 °C and 4 °C acclimated animals, resting metabolic rate was measured in awake animals at 30 °C. In addition, NE-stimulated (1 mg NE/kg body weight in saline, (−) arterenol bitartrate, intraperitoneally administered) energy expenditure was evaluated in anaesthetized (pentobarbital, 90 mg/kg) animals at 33 °C. Animals were allowed to recover from NE administration for 2–3 h at 30 °C. The mice where then rehoused at their acclimatisation temperature for 1 wk prior to tissue collection for gene and protein expression analysis to avoid effects of NE on gene expression. Oxygen consumption in conscious animals was followed for 3 h using an open circuit system with a chamber volume of 3 l and a flow rate of 1 l/min (Somedic, Hörby, Sweden). This system allowed the ambient temperature of the instruments to be adjusted between 5 °C and 40 °C, together with the volume and flow rates to optimise the system for the particular investigation. Oxygen consumption, carbon dioxide release, and ambient temperature data were collected every second minute via MacLab/2e (AD Instruments Pty. Ltd., Castle Hill, Australia). Resting metabolic rate was defined as the average of the lowest metabolic rates observed at three time points. Determinations in WT and PGC1βKO mice were performed in alternating order.

### Catherization and dobutamine treatment.

Mice were anesthetized with isoflurane and underwent endotracheal intubation. The airway was connected to mouse ventilator (Model 687, Harvard Apparatus, Holliston, Massachusetts, United States) to control breathing. The oxygen flow rate was 1 l/min. The left jugular vein was identified and accessed by cut down method using a 25 G needle connected to a syringe with dobutamine hydrochloride (Sigma) that was mounted on a Standard Infuse/Withdraw Harvard 33 Twin Syringe Pump (Harvard Apparatus). A micromanometer-tipped catheter (Millar Instruments, Houston, Texas, United States) was then inserted into the left ventricle via right carotid artery, and hemodynamic measurements was obtained as described [28]. After obtaining baseline left ventricular pressure and heart rate readings, the dobutamine infusion was commenced. The initial infusion rate was 10 ng/min/g BW, with hemodynamic recordings taken at 2, 4, and 6 min. The infusion rate was then increased to 40 ng/min/g BW, and additional readings were obtained at 2, 4, and 6 min after the dose adjustment.

### Tissue collection and RNA extractions.

Mice were anaesthetised using isoflourane. Blood, tissues used for RNA, protein extraction, and histology were prepared as previously published [26,29].

### Biochemical analysis.

Enzymatic assay kits were used for determination of basic blood biochemical parameters as described in [26,29]. The size distribution profiles of serum lipoproteins were measured in pooled plasma samples using a high-performance liquid chromatography system (HPLC), SMART, and a Superose 6 PC 3.2/30 column as described before [30].

### Histological sample preparation and analysis.

Tissue samples for morphological analysis were prepared according to published protocols [29]. For light microscopy, sections were stained with haematoxylin and eosin. For adipose tissue, images of each section were acquired using a digital camera and microscope (Olympus BX41, Olympus Corporation, Tokyo, Japan) and adipocyte area was measured using AnalySIS software (Soft Imaging System, Münster, Germany). Two fields from each section from gonadal, subcutaneous, and omental adipose tissue depots (*n* = 7–8 mice per genotype) were analysed to obtain the mean cell area per animal. For preparation of BAT, soleus muscle, and hearts for electron microscopy, mice were exsanguinated by perfusion with physiological saline containing 0.1% sodium nitrate until no blood was left. Mice were then perfused with 60–90 ml of fixative (3% glutaraldehyde and 1% formaldehyde in 0.1 mol/l 1,4-piperazine diethane sulfonic acid (PIPES) buffer (pH 7.4) containing 2 mol/l calcium chloride). Isotropic uniform random planes of section through the left ventricular wall in hearts were prepared using the orientator principle as described in [31]. Soleus samples were prepared at equal lengths along the long axis of the muscle. The blocks of tissue were sectioned in small fragments with a razor blade to <1 mm in one dimension and fixed by immersion at 4 °C for a further 3–4 h. Samples were then washed in 0.1 M PIPES and post-fixed in 1% osmium tetroxide, dehydrated in acetone, and embedded in Spurr's epoxy resin. Thin sections were obtained with a Leica UCT ultramicrotome (Leica Microsystems, Milton Keynes, United Kingdom) and examined with a transmission electron microscope (CM100, Philips, Netherlands).

### Stereological assessment of mitochondria.

Stereological assessments of mitochondrial volume fractions (*V*_v_) and the surface density (*S*_V_) of their inner and outer membranes in heart were performed as described in [31] and in [32].

### Permeabilised tissue and isolated mitochondrial respiration studies.

Isolation of mitochondria was performed in hearts and skeletal muscle from 10-wk-old male PGC-1β mice and their WT littermates. Mice were killed by cervical dislocation. The hearts or hind limb skeletal muscle of four mice per genotype were pooled and immediately placed in ice-cold isolation medium (for hearts, 250 mM sucrose, 5 mM Tris, 2 mM EGTA at pH 7.4; for skeletal muscle, 100 mM KCl, 50 mM Tris, 2 mM EGTA at pH 7.4). Mitochondria were prepared essentially as described in [33]. Mitochondrial respiration was assessed in saponin-skinned soleus fibres prepared as in [14,34].

### Measurement of proton conductance and ETC function in isolated mitochondria.

The kinetics of proton conductance and ETC function were measured in mitochondria in the presence of oligomycin (1 μg/ml), where that rate of respiration is directly proportional to the leak of protons across the mitochondrial inner membrane rather than a combination of ADP phosphorylation and proton leak. Mitochondrial function was assessed as described previously [35,36].

### Quantitative RT-PCR analysis of gene expression.

Total RNA was isolated from tissues as described and reverse-transcribed using the SuperscriptII kit (Invitrogen, Frederick, Maryland, United States) or Hi-Capacity cDNA archive kit (Applied Biosystems, Foster City, California, United States), following the manufacturers protocol. Oligonucleotide primers and TaqMan probe were designed using Primer Express, version 2.0 (Applied Biosytems). Primer and probe sequences, together with gene abbreviations, can be found in Tables S4–S6.

### Microarray analysis.

Male 14-wk-old mice were killed and dissected as above. The tissues were extracted for RNA as above and purified using the RNA clean-up protocol from the RNeasy Mini Kit (Qiagen Ltd, Crawley, UK). RNA was quantified spectroscopically at 260 nm using a GeneQuant Nucleotide calculator (Amersham Biosciences, Little Chalfont, UK) and checked for integrity on a 1% TBE gel using ethidium bromide staining. cDNA was amplified from total RNA using template-switching PCR and labeled with Cy3 or Cy5 dyes as previously described [37]. Binomial pathway enrichment analysis was then performed. A more detailed protocol can be found in Protocol S1. Raw data from the analysis are presented in Dataset S1 (pathway comparison) and Dataset S2 (raw data from the chip analysis).

### Mitochondrial isolation, protein extraction, and Western analysis.

Female 15-wk-old chow-fed mice were anaesthetised using isoflurane and killed by cervical dislocation and heart removal. BAT, heart, and soleus muscle tissue was removed and either snap-frozen in liquid nitrogen or used to prepare a mitochondrial extract as described in Protocol S1. In general, 10 μg of total lysate or 3 μg of mitochondrial preparation was separated by SDS-PAGE on 14% gels. After transfer, the membranes were cut and placed in blocking buffer (5% powdered milk in 1× PBS and 0.1% Tween-20). We used anti-OxPhos complex I (α-subcomplex 9) and anti-OxPhos complex III (Fe-S core protein) (Molecular Probes, Carlsbad, California, United State) and anti-succinate dehydrogenase subunit B, anti-Cox4, and anti-ATP synthase β-subunit from Abcam, Cambridge, UK. All primary antibodies were used at ratios of 1:2,000, except anti-Cox4, which was used at 1:20,000. Goat anti-mouse horseradish peroxidase secondary antibody (Pierce, Rockford, Illinois, United States) was used at 1:40,000 for all blots, the membranes were reassembled, and the proteins detected using the ECL-Plus system (Amersham Biosystems). Films were scanned and analysed using NIH Image 1.34s.

### Statistical analysis.

The data presented here were analysed using the Student *t*-test or analysis of variance (ANOVA) with post-hoc tests on the program StatView Version 4.5 (Abacus Concepts, Berkley, California, United States). Data are presented as mean ± SEM unless stated. *p* < 0.05 was considered significant. The significance levels displayed on figures are as follows: * indicates *p*< 0.05, ** indicates *p* < 0.01, *** indicates *p* < 0.001.

## Supporting Information

Dataset S1 Data Used for the Comparative Pathway Analysis of BAT, Heart, and Liver Tissues Using KEGG and KEGG2 Pathways (62 KB XLS)

Dataset S2 Raw Data Text Files from the Spotted Array Experiments on Heart, Liver, and BAT Used in the Comparative Pathway Analysis (3.2 MB XLS)

Figure S1 Size Distribution of Adipocytes from WT and PGC1βKO Mice Two fields from each section from (A) omental WAT, (B) subcutaneous WAT, and (C) female gonadal WAT depots (age 24 wk, *n* = 7–8 mice per genotype) were analysed to obtain the mean cell area per animal.
(43 KB PPT)

Figure S2 Histological Sections of Tissues Sections are as follows: BAT (A and B), liver (C and D), soleus muscle (E and F), pancreatic sections stained for glucagon (G and H), or pancreatic sections stained for insulin (I and J). A representative section from WT (A, C, E, G, and I) or PGC1βKO (B, D, F, H, and J) are shown, *n*=6.
(1.7 MB PDF)

Figure S3 Deletion of PGC-1β Does Not Affect Insulin Sensitivity Mice were placed on normal diet or HFD for 13 wk. GTTs (left panels) or ITTs (right panels) were performed on the mice at 16 wk of age. Plasma glucose levels of WT mice (open circles) and PGC1βKO (solid circles) were measured during the protocol.
(115 KB PPT)

Figure S4 Expression of mRNAs Assessed on Soleus from 12-wk-Old Male WT (Black Bars) and PGC1βKO (White Bars) Mice Individual measurements were standardised using 18S, and the average of the WT group set to 1. *n* = 6 mice per group.
(34 KB PPT)

Figure S5 Assessment of Isolated Soleus Mitochondrial Function from WT and PGC1βKO Mice Soleus tissue was pooled from four WT mice (black bars) or four PGC1βKO mice (white bars) and mitochondria isolated on five separate occasions (*n* = 5). Respiration states (A) were calculated using oxygen consumption rates in isolated mitochondria energised with succinate (4 mM). Respiratory control ratios (B) were determined for mitochondria as state 3 divided by state 4. Proton leak kinetics (C) were measured as described under Materials and Methods.
(35 KB PPT)

Figure S6 PGC-1β Ablation Results in Few Changes in Gene Expression in Liver Gene expression was analysed in 12-wk-old fed-state male WT (black bars) and PGC1βKO (white bars) livers. Individual measurements are standardised using 18S and then the average of the WT group was set to 1. *n* = 6 mice per group.
(36 KB PPT)

Figure S7 Gene Expression of PGC-1α and PGC-1β (A) and for a Selection of Genes Involved with Fatty Acid and Cholesterol Handling in the Liver (B) in 8-wk-Old Female Mice Fed Surwit Diet for 24 h WT chow, black bars; WT Surwit HFD, white bars; PGC1βKO chow, dark gray bars; PGC1βKO Surwit HFD, light gray bars. Individual measurements are standardised using 36B4 and then the average of the WT chow group was set to 1. *n* = 4–7 per condition.
(35 KB PPT)

Protocol S1 Supplementary Protocols (99 KB DOC)

Table S1 WT and PGC1βKO Mouse Parameters for Energy Intake and Faecal Output over 48 h The food intake and energetic content of faeces from 12-wk-old male mice was determined over a 48-h period. *n* = 5–8 per group.
(21 KB DOC)

Table S2 WT and PGC1βKO Tissue Weights in 14-wk-Old Male Mice The data are presented both comparing raw weights and as tissue weight/body weight. Statistical comparison between WT and PGC1βKO mice was performed using *t*-tests. *n* = 6–8 per group. *p* values are shown where *p* is less than or equal to 0.2.
(25 KB DOC)

Table S3 Comparison of KEGG Pathways Significantly Down-regulated in all Three Arrayed Tissues (Liver, BAT, and Heart) RNA was isolated from 14-wk-old normal diet-fed male PGC1βKO and WT mice and then analysed using spotted arrays as described in the Materials and Methods. Data from these chips were then analysed using for pathway changes using the KEGG pathway database. Pathways with only one changed gene were discounted. Data are displayed as pathway name followed by *p* value for the binomial test. A pathway *p* value of <0.05 was considered significant.
(22 KB DOC)

Table S4 Sequences for Taqman Primers and Probes Sybr as a probe indicates the use of a Sybr-Green system instead of Taqman.
(31 KB DOC)

Table S5 Sequences for Taqman Primers and Probes Sybr as a probe indicates the use of a Sybr-Green system instead of Taqman.
(29 KB DOC)

Table S6 Sequences for Taqman Primers and Probes Sybr as a probe indicates the use of a Sybr-Green system instead of Taqman.
(29 KB DOC)

## Abbreviations

BAT: brown adipose tissue
Cox: cytochrome oxidase subunit
ES cell: embryonic stem cell
ETC: electron transport chain
HDL: high-density lipoprotein
HFD: high-fat diet
KEGG: kyoto encyclopedia genes and genomes
LDL: low-density lipoprotein
NE: norepinephrine
NS: not significant
OxPhos: oxidative phosphorylation
PGC: peroxisome proliferator-activated receptor-gamma coactivator
RER: respiratory exchange ratio
VLDL: very low-density lipoprotein
WAT: white adipose tissue
WT: wild-type
