# Supplementary material for: Ablation of PGC-1β Results in Defective Mitochondrial Activity, Thermogenesis, Hepatic Function, and Cardiac Performance
Source: PLoS Biol. 2006 Nov 7;4(11):e369. doi: 10.1371/journal.pbio.0040369 (PMC1634886; doi:10.1371/journal.pbio.0040369)
Supplement: Supplementary file 2 [file pbio.0040369.sd003.doc]

**Ablation of PGC-1β Results in Defective Mitochondrial Activity, Thermogenesis, Hepatic Function and Cardiac Performance**

# Supplementary Protocols S1

# Christopher J. Lelliott^1,2 *^, Gema Medina-Gomez^1*^, Natasa Petrovic^3^, Adrienn Kis^1^, Helena M. Feldmann^3^, Mikael Bjursell^2^, Nadeene Parker^1^, Keira Curtis^1^, Mark Campbell^1^, Ping Hu^4^, Dongfang Zhang^4^, Sheldon E. Litwin^4^, Vlad G. Zaha^5^, Kimberly T. Fountain^5^, Sihem Boudina^5^, Mercedes Jimenez-Linan^1^, Margaret Blount^1^, Miguel Lopez^1^, Aline Meirhaeghe^6^, Mohammad Bohlooly-Y^2^, Leonard Storlien^2^,Maria Strömstedt^2^, Michael Snaith^2^, Matej Orešič ^6^, E. Dale Abel^5^, Barbara Cannon^3^, Antonio Vidal-Puig^1^

# ^1^ Department of Clinical Biochemistry, University of Cambridge, Addenbrooke’s Hospital, Hills Road, Cambridge CB2 2QR, UK

# ^2^ AstraZeneca R&D, SE-431 83 Mölndal, Sweden.

# ^3^ The Wenner-Gren Institute, The Arrhenius Laboratories F3, Stockholm University, SE-106 91 Stockholm, Sweden

^4^ Division of Cardiology, University of Utah, 30 N. 1900 E. Salt Lake City, UT 84132, USA

# ^5^ Division of Endocrinology, Metabolism and Diabetes and Program in Human Molecular Biology & Genetics, University of Utah 15 N 2030 E, Bldg 533, Room 3410B, Salt Lake City, UT 84112, USA

^6^ INSERM, U508, Institut Pasteur de Lille, 1 rue Calmette, 59019 Lille Cedex, France.

# ^7^ VTT Technical Research Centre of Finland, Tietotie 2, Espoo, P.O. Box1500, FIN-02044, Finland

**^*^ These authors contributed equally to this work.**

# Running Head: Phenotype of the PGC-1β knockout mouse

**Corresponding author**:

Antonio Vidal-Puig,

Department of Clinical Biochemistry

University of Cambridge

Addenbrooke’s Hospital

Hills Road, Cambridge

CB2 2QR, UK

Email: [ajv22@cam.ac.uk](mailto:ajv22@cam.ac.uk)

Tel: 01223762790

# Fax: 44-1223-330598

# Supplementary Materials and Methods

# Materials and reagents

### All reagents used in this paper were supplied from Sigma-Aldrich (St. Louis, MO), unless stated.

### Generation of PGC1βKO mice

A triple LoxP strategy was used to target the *PGC-1β* locus in order to generate mice with both standard and conditional KO alleles at this locus. The region of homology used in the targeting vector was amplified by proof-reading PCR using a 129/SvJ genomic RPCI Mouse PAC clone as template. In essence, the targeting vector was a ~8kb 129/SvJ mouse genomic subclone containing a floxed neomycin phosphotransferase selectable marker cassette inserted into intron 3 and a single LoxP site inserted into intron 5 (Figure 1A). After linearization, the targeting construct was electroporated into R1 ES cells (derived from 129/SvJ) and neomycin-resistant clones were selected in G-418-containing (300 µg/ml) media. Of 426 G418-resistant clones screened, one targeted clone was identified using a PCR with a primer located outside the short arm combined with a primer to the promoter region of the neomycin cassette. Further PCR screens and Southern analyses confirmed the fidelity of the gene targeting in this ES clone (for an example, Figure 1B and C). The clone was expanded and injected into C57Bl/6 blastocysts to generate chimeric mice. Chimeric males were crossed to C57Bl/6 females and genotyping of the *agouti* offspring was performed from tail biopsies. The following primers were used to identify offspring with the targeted allele: A forward primer in intron 3 within the short arm homology sequence 5´-ggacaaagaggaagtcagctgga-3´ combined with a reverse neo-specific primer 5´-gctgcctcgtcctgcagttcat-3´. Heterozygous triple LoxP mice were then bred to ROSA26Cre [1] mice in order to generate heterozygous PGC-1β KO mice which had undergone Cre-mediated deletion of the intervening region of DNA between the outermost LoxP sites. Such mice were identified using the following genotyping primers: Forward, 5´-gcacacccgtgaatactatgta-3´; Reverse 1, 5´-ccttgggcctccatctctgtt-3´ and Reverse 2, 5´-caaggagcaggaactgggatt-3´. These primers gave a product of approximately 0.5kb for the Cre-recombined allele and products of approximately 2.8 and 0.6kb with the wild-type allele (Figure 1D). Heterozygous PGC-1β KO mice were then intercrossed to generate mice homozygous for the PGC-1β deletion. To verify that the deletion of *PGC-1β* coding sequences resulted in a null mutation, total RNA was prepared from heart and skeletal muscle of homozygous, heterozygous, and wild type littermates using the RNA STAT-60 Kit according to the manufacturer’s instructions (Tel-Test Inc, Friendswood, Texas). cDNA was synthesized using Superscript^TM^ II RNase H- Reverse Transcriptase and random hexamer primers (Invitrogen, Frederick, Maryland) to demonstrate that exons 4 and 5 had been deleted in the PGC1βKO mice. Mice used in this paper were backcrossed between 3 and 6 times to a C57BL6/J background. Littermate controls were used for all experiments. .

# Long Term Feeding and Growth Studies

Mice were placed at weaning (3 weeks old) on normal chow diet (12% fat, 62% carbohydrates, and 26% protein with a total energy content of 12.6 kJ/g (R3 diet, Lactamin AB, Stockholm, Sweden). Mouse weights were taken at the same time each week, until the end of the specific protocol period. Mice were routinely housed at 22°C, except for those used for the BAT activity and cold acclimatisation experiments (see below).

**Glucose (GTT) and Insulin tolerance tests (ITT) on chow and high fat diet fed mice** were performed as previously described [2]. Mice were placed on either chow or high fat diet (HFD) (45% of calories derived from fat; D12451, Research Diets, New Brunswick, NJ) for 13 weeks. Food was removed over night before the initiation of a glucose tolerance test (GTT, 1 g of glucose/kg of body weight) or 6 hours prior to the insulin tolerance test (ITT, 0.75 U/kg insulin) [3]. Blood glucose levels were monitored using a Onetouch Ultra glucose meter (LifeScan, High Wycombe, UK) from a 2.5µl of tail blood at times indicated after glucose administration. Insulin was measured as in [2].

**Acute dietary measurements and interventions**

To examine 48h food intake, cages (23 × 16 cm) were prepared with normal chow and incubated at 80°C for 1h to correct for any differences in humidity. After 2h at room temperature the cages were accurately weighed. 12h-fasted mice were put in preweighed cages with free access to food and water. After 48h, the mice were removed and all faecal matter was collected. The cages were reincubated at 80°C in order to dry out waterspill and urine, and reweighed after 2 h cooling. The difference in weights of the cage before and after the 48h assessment produced the weight of food consumed. For measurement of energy content of faeces and food, samples were dried at 55°C overnight and stored in airtight containers at -20°C until assayed. The gross energy content of the dried samples was determined using a bomb calorimeter (C5000, IKA^®^ Werke GmbH & Co. KG, Germany). To assess the effect of 24h high-fat feeding, 8 week-old female mice were assigned into two groups: *ad libitum* normal food or *ad libitum* Surwit diet (58% of calories derived from fat, predominantly hydrogenated coconut oil; D12331, Research Diets, New Brunswick, NJ). Start of the 24h period was 9am. At the end of the time period, mice were killed and dissected as above. Water was freely available during all procedures.

# Body composition and indirect calorimetry

For body composition analysis, Dual Energy X-ray absorptiometry (DEXA, Lunar Corporation) was performed on isoflurane anaesthetized mice. Images were analysed by densitometry using PIXImus imager (Lunar GE Medical Systems, Madison, WI, USA). Oxygen consumption (VO_2_) and carbon dioxide production (VCO_2_) were measured using an open circuit calorimetry system (Oxymax; Columbus Instruments International, Columbus, OH) [4]. The animals were placed in calorimeter chambers with *ad libitum* access to normal lab diet and water for 48 h. An air sample was withdrawn for 75s every 20 min. The O_2_ and CO_2_ content were measured by a paramagnetic oxygen sensor and a spectrophotometric CO_2_ sensor. These values were used to calculate VO_2_ and RER. The value of metabolic rate was correlated to individual body weights and to values of lean mass obtained by DEXA analysis. The resting and maximum spontaneous metabolism was analysed by averaging the three lowest and highest recorded VO_2_ respectively values during the final 24h of the procedure. The reported RER was calculated as the average RER measurement during the final 24h of the procedure.

# Cold acclimatisation and BAT activity

PGC1βKO mice and WT littermates were exposed to 30°C for three weeks, placed at 18°C for one week and then exposed to 4°C for 3 weeks or kept at 30°C for the duration of the protocol. For both 30°C and 4°C acclimated animals, resting metabolic rate was measured in awake animals at 30°C. In addition, norepinephrine (NE)-stimulated (1mg/kg body weight in saline, (-) Arterenol bitartrate, i.p. administered) energy expenditure was evaluated in anaesthetized (pentobarbital, 90 mg/kg) animals at 33°C. Animals were allowed to recover from NE administration for 2-3hours at 30°C. The mice where then rehoused at their acclimatisation temperature for 1 week prior to tissue collection for gene and protein expression analysis, to avoid effects of NE on gene expresson. Oxygen consumption in conscious animals was followed for 3h, using an open circuit system with a chamber volume of 3 litres and a flow rate of 1 l/min (Somedic, Hörby, Sweden). This system allowed the ambient temperature of the instruments can be adjusted between 5°C and 40°C, together with the volume and flow rates to optimise the system for the particular investigation. Oxygen consumption, carbon dioxide release and ambient temperature data were collected every second minute via MacLab/2e (AD Instruments Pty. Ltd., Castle Hill, Australia). Resting metabolic rate was defined as the average of the lowest metabolic rates observed at three time points. Determinations in WT and PGC1βKO mice were performed in alternating order. Curves presented are the means ± standard error (*n*=5/group).

# Catherization and dobutamine treatment

Mice were anesthetized with isoflurane and underwent endotracheal intubation. The airway was connected to mouse ventilator (Model 687, Harvard Apparatus, Holliston, MA) to control respiration. The oxygen flow rate was 1l/min. The left jugular vein was identified and accessed by cut down method using a 25G needle connected to a syringe with Dobutamine hydrochloride (Sigma) that was mounted on a Standard Infuse/Withdraw Harvard 33 Twin Syringe Pump (Harvard Apparatus). A micromanometer-tipped catheter (Millar Instruments, Houston, TX) was then inserted into the left ventricle via right carotid artery, and hemodynamic measurements obtained as described [5]. After obtaining baseline left ventricular pressure and heart rate readings, the dobutamine infusion was commenced. The initial infusion rate was 10ng/min/g body wt, with hemodynamic recordings taken at 2, 4 and 6 minutes. The infusion rate was then increased to 40ng/min/g body wt and additional readings obtained at 2, 4 and 6 minutes after the dose adjustment.

# Tissue collection and RNA extractions

# Mice were anaesthetised using isoflurane. Blood was taken by cardiac puncture, centrifuged at 1000 x *g* for 5 minutes at 4°C and serum stored at –80°C until analysis [6]. Tissues used for RNA and protein extraction were excised, weighed and snap-frozen in liquid nitrogen, for subsequent storage at -80ºC. Samples for histology were placed in formalin fixative and stored at room temperature prior to sectioning. RNA extractions from tissues were performed using Trizol Reagent (Invitrogen) as per the manufacturer’s instruction, but using two rounds of purification with Trizol/chloroform prior to isopropanol precipitation. RNA concentration was assessed using a NanoDrop ND-1000 Spectrophotometer (NanoDrop Technologies, Wilmington, DE) or GeneQuant Nucleotide Calculator (Amersham Biosciences, Chalfont St. Giles, UK).

**Biochemical analysis**

Enzymatic assay kits were used for determination of plasma free fatty acids (Roche, Lewes, UK), cholesterol (Roche) and total triglycerides (Sigma). Elisa kits were used for measurements of Leptin (R & D Systems, Oxford, UK), Insulin (DRG Diagnostics International, Mountainside, NJ) and Adiponectin (B-Bridge International, Mountain View, CA) according to manufacture’s instructions. The size distribution profiles of serum lipoproteins were measured in pooled plasma samples using a high-performance liquid chromatography system (HPLC), SMART, and a Superose 6 PC 3.2/30 column as described before [7].

**Histological sample preparation and analysis**

Tissue samples for morphological analysis were prepared accordingly to published protocols [6,8]. For light microscopy, sections were stained with haematoxylin and eosin.

For adipose tissue, images of each section were acquired using a digital camera and microscope (Olympus BX41) and adipocyte area was measured using AnalySIS software (Soft Imaging System). Two fields from each section from gonadal, subcutaneous and omental adipose tissue depots (n=7-8 mice/genotype) were analysed to obtain the mean cell area per animal. For preparation of BAT, soleus muscle and hearts for electron microscopy, mice were exsanguinated by perfusion with physiological saline containing 0.1% sodium nitrate until no blood was left and then perfused with 60 - 90 ml of fixative (3% glutaraldehyde and 1% formaldehyde in 0.1 mol/L 1,4-piperazine diethane sulfonic acid (PIPES) buffer (pH 7.4) containing 2 mol/L calcium chloride). Isotropic uniform random planes of section through the left ventricular wall in hearts were prepared using the orientator principle as described in Howard and Reed [9]. Soleus samples were prepared at equal lengths along the long axis of the muscle. The blocks of tissue were sectioned in small fragments with a razor blade to < 1mm in one dimension and fixed by immersion at 4 degrees for a further 3-4 hours. Samples were then washed in 0.1M PIPES and post-fixed in 1% osmium tetroxide, dehydrated in acetone and embedded in Spurr’s epoxy resin. Thin sections were obtained with a Leica UCT ultramicrotome (Leica Microsystems, Milton Keynes, UK) and examined with a transmission electron microscope (CM100, Philips, The Netherlands).

# Stereological assessment of mitochondria

Stereological assessments of mitochondrial volume fractions (V_v_) and the surface density (S_V_) of their inner and outer membranes in heart were performed as described in Howard and Reed [9]. The point counting method was used to estimate volume fraction of mitochondria in the heart. Images captured at a magnification of 44000x were overlain with a quadratic lattice and the number of intersections overlying mitochondria was counted. The surface density of the outer and inner mitochondrial membranes cristae was then measured as in [10] and described in Howard and Reed [9]. In brief, images of mitochondria were overlain with a curvilinear lattice and surface density was estimated using the formula: S_v_ = 2 x (i/L), where I = the number of intercepts of test lines with either, outer or inner mitochondrial membranes and L = length of test line falling on all mitochondrial compartments.

# Permeabilised tissue respiration studies

Mitochondrial respiration was assessed in saponin-skinned soleus fibres prepared as in [11,12]. Respiration was measured at 25°C using an optical probe (Oxygen FOXY Probe, Ocean Optics, Dunedin, Florida, USA). The substrate for the soleus fibres was 5mM succinate and 10μM rotenone. Basal respiration rates before the addition of ADP (V_0_) were defined as State 2. Maximally ADP (1 mmol/L)-stimulated respiration rates (V_ADP_) were defined as state 3, and respiration rates without ADP phosphorylation and with 1μg/ml oligomycin (V_oligomycin_) was defined as State 4 as stated in [13]. Oxygen consumption was expressed as nmol of O_2_ · min^-1^ · mg dry fibre weight^-1^. ATP concentration was determined using a bioluminescence assay based on the luciferin/luciferase reaction with the ATP assay kit (ThermoLabsystems, Waltham, MA).

# Isolation of mitochondria and measurement of oxygen consumption

Isolation of mitochondria was performed in hearts and skeletal muscle from 10 week–old male PGC-1β and their WT littermates. Mice were killed by cervical dislocation. The hearts or hind limb skeletal muscle of 4 mice per genotype were pooled and immediately placed in ice-cold isolation medium (For hearts, 250 mM sucrose, 5 mM Tris, 2 mM EGTA; for skeletal muscle, 100 mM KCl, 50 mM Tris, 2 mM EGTA at pH 7.4). Mitochondria were prepared essentially as described [14]. Briefly, tissues were minced and incubated with 2500 U/L protease (with skeletal muscle media having additional supplement of 0.5 % BSA, 5 mM MgCl_2_, 1 mM ATP) for 3 mins, followed by homogenisation using a Dounce homogeniser. Cell debris was removed at 700 x *g* (heart) or 490 x *g* (skeletal muscle) and the mitochondrial pelleted at 8500 x *g* (heart) or 10368 x *g* (skeletal muscle). Pellets were then resuspended and washed three times in isolation media. Protein concentration was determined by the DC protein assay (Bio-Rad, Hercules, CA). Oxygen consumption was measured at 37 °C using a Clarke-type oxygen electrode (Rank Brothers Ltd., United Kingdom). Mitochondria (0.35 mg/ml) were incubated in air-saturated assay medium (120 mM KH_2_PO_4_, 3 mM HEPES, 1 mM EGTA, and 0.3% (w/v) defatted BSA, pH 7.2) that was assumed to contain 406 nmol of oxygen/ml at 37 °C. Electrode linearity was checked periodically by following mitochondrial respiration in the presence of 0.2μM FCCP from 100% to 0% air saturation. Mitochondrial oxygen consumption was measured in assay medium containing 5μM rotenone, 4mM succinate (state 2), 250μM ADP (state 3, natural state 4 once depleted) plus 1 μg/mg oligomycin (oligomycin state 4) then 1μM FCCP. Respiratory control ratios (RCRs) were calculated as the state 3 respiration rate divided by the state 4 respiration rate.

# Measurement of proton conductance and electron transport chain function in isolated mitochondria

The kinetics of proton conductance and electron transport chain function were measured in mitochondria in the presence of oligomycin (1μg/ml) where that rate of respiration is directly proportional to the leak of protons across the mitochondrial inner membrane rather than a combination of ADP phosphorylation and proton leak. Mitochondrial function was assessed using simultaneous measurements from an oxygen electrode (see above) and an electrode sensitive to the membrane potential-dependent probe, TPMP^+^, essentially as described previously [15,16]. Mitochondria were incubated in assay medium containing 5μM rotenone, 1μg/ml oligomycin, and 80 ng/ml nigericin. The potential electrode was calibrated using sequential additions of TPMP up to 2.5μM TPMP then 4mM succinate was added to start the reaction. Rate of proton leak across the mitochondrial inner membrane is a function of membrane potential and so kinetics of proton conductance were measured by varying membrane potential using titrations of the ETC inhibitor malonate (up to 1 mM). A further 0.1μM FCCP was added at the end of each run to dissipate membrane potential and release TPMP back into the medium to allow for drift in the electrode. Similarly, as the rate of electron transport is a function of proton leak, the kinetics of the electron transport chain were measured by adding titrations of a mitochondrial uncoupler, FCCP (up to 1μM). A TPMP-binding correction was taken to be 0.4 per μl/mg protein [17].

**Quantitative RT-PCR analysis of gene expression**

Total RNA was isolated from tissues as described above and reverse-transcribed using the SuperscriptII kit (Invitrogen) or Hi-Capacity cDNA archive kit (Applied Biosystems, Foster City, CA), following the manufacturers protocol. Oligonucleotide primers and TaqMan probe were designed using Primer Express, version 2.0 (Applied Biosytems). Primer and probe sequences, together with gene abbreviations, can be found in Supplementary Tables 4, 5 and 6. Primers and probe for 18S are Applied Biosystems proprietary sequences. The TaqMan probes were labelled at the 5’ end with the reporter dye FAM (6-carboxy-fluorescein) and at the 3’ end with the quencher TAMRA (6-carboxy-tetramethyl-rhodamine). Oligonucleotide primers and TaqMan probes were purchased from Applied Biosystems and Sigma-Genosys. For reactions using the Taqman system, PCR was carried out on ABI 7700 and 7900HT sequence detection systems (Applied Biosystems) using the following standard conditions: 50°C for 2 min, 95°C for 10 min followed by 40 cycles of 95°C for 15 sec, 60°C for 1 min. Several targets were detected using the Sybr system, (Applied Biosytems) which followed the manufacturer’s instructions for use. The Ct value for each sample was compared to a standard curve in order to define an arbitrary expression level. The value was then standardised to an internal control (18S or 36B4) and then compared to the WT control group whose value was set to 1.

# Microarray analysis

14 week-old male mice were killed and dissected as above. The tissues were extracted for RNA as above and purified using the RNA clean-up protocol from the RNeasy Mini Kit (Qiagen Ltd, Crawley, UK). RNA was quantified spectroscopically at 260nm using a GeneQuant Nucleotide calculator (Amersham Biosciences, Little Chalfont, UK) and checked for integrity on a 1% TBE gel using ethidium bromide staining. cDNA was amplified from total RNA using template-switching PCR and labelled with Cy3 or Cy5 dyes as previously described [18]. The labelled products were purified on an AutoSeq G-50 column; the Cy5 and Cy3 samples were then pooled and ethanol precipitated. The samples were hybridised to Compugene Mouse Known Gene set of 7,524 oligonucleotides, (supplied by MRC-HGMP, Hinxton, UK). For each set of conditions tested, duplicate and dye-swap hybridisations were performed. Labelled targets were resuspended in 30μl of hybridisation buffer (40% formamide, 5xSSC, 5xDenhardt's solution, 1mM sodium pyrophosphate, 50mM Tris pH 7.4, 0.1% SDS) together with 2μg mouse Cot1 DNA (Invitrogen), denatured at 95°C for 5 min, incubated at 50°C for 5 min and then centrifuged at 13,000 r.p.m. for 5 min before being applied to the arrays. Hybridisations were performed under a coverslip at 50°C in a humidified chamber for 16 h. Following hybridisation, slides were washed twice in 2xSSC for 10 min, twice in 0.1x SSC/0.1% SDS for 5 min and finally twice in 0.1x SSC for 5 min; all washes were performed at room temperature. After washing, slides were dried by centrifugation at 2000 x *g* for 3 min. Arrays were scanned on an Agilent G2565 scanner according to manufacturer’s instructions. Raw image data were extracted using Imagene^TM^ 5.0 software (BioDiscovery). Data were imported into GeneSpring^TM^ 6.2 (Silicon Genetics ) for analysis. Normalization was performed using the Loess algorithm. Identification of genes with a ratio statistically significantly different from 1 (the average of the WT group values) was performed using a *t*-test at the 95% confidence level. Pathway enrichment analysis were performed by a ‘hit-counting’ (binomial) method which matches a list of selected genes (1.5 fold upregulated) to a pathway [19]. The resulting p-value describes the enrichment of the pathway in the list of genes. Pathways and gene sets were obtained from KEGG [20]. Only pathways that contained 2 or more hits in each tissue were included for analysis. Raw Data from the analysis is presented in Dataset S1 (Pathway comparison) and Dataset S2 (Raw data from the chip analysis).

**Mitochondrial isolation, protein extraction and Western analysis.**

15 week-old chow-fed female mice were anaesthetised using isoflurane and killed by cervical dislocation and heart removal. BAT, heart and soleus muscle tissue was removed and either snap-frozen in liquid nitrogen or used to prepare a mitochondrial extract. For the mitochondrial extract, tissues were placed in STE buffer (250mM Sucrose, 5mM Tris-HCl, 2mM EGTA, pH 7.4 with 1 tablet Roche Mini Complete Protease Inhibitors/10ml buffer) and homogenised using a teflon-glass homogeniser. The homogenate was then centrifuged at 700 x g for 5 minutes at 4ºC. The supernatant was then removed and centrifuged again at 8000 x g for 10 minutes at 4ºC. The pellet from this was then frozen on dry ice and stored at –80ºC. When required, the pellets were resuspended in 10mM Hepes pH7.2, 1mM EDTA with protease inhibitor. For tissue lysates, tissues were crushed under liquid nitrogen, the powder collected and resuspended in RIPA buffer (1X PBS, 1% NonIdet-P40, 0.5% sodium deoxycholate, 0.1% SDS with 1 tablet Mini Complete Protease Inhibitors/10ml buffer). These were then further homogenised using a hand-held Polytron. The homogenates were then centrifuged at 14,000 x g for 30 minutes at 4ºC. The supernatant was removed for analysis. Protein concentration was determined using the BCA Protein Determination Kit (Pierce, Cramlington, UK). In general, 10μg of total lysate or 3μg of mitochondrial preparation was separated by SDS-PAGE on 14% gels. After transfer, the membranes were cut and placed in blocking buffer (5% powdered milk in 1X PBS and 0.1% Tween-20). We used anti-OxPhos complex I (α-subcomplex 9) and anti-OxPhos complex III (Fe-S core protein) from Molecular Probes, Carlsbad, CA; anti-succinate dehydrogenase subunit B, anti-Cox4 and anti-ATP synthase β-subunit from Abcam, Cambridge, UK. All primary antibodies were used at 1:2000, except anti-Cox4, which was used at 1:20000. Goat anti-mouse horseradish peroxidase secondary antibody (Pierce) was used at 1:40000 for all blots, the membranes were reassembled and the proteins detected using the ECL-Plus system (Amersham Biosystems). Films were scanned and analysed using NIH Image 1.34s.

1. Soriano P (1999) Generalized lacZ expression with the ROSA26 Cre reporter strain. Nature Gen 21: 70-71.

2. Medina-Gomez G, Virtue S, Lelliott C, Boiani R, Campbell M, et al. (2005) The link between nutritional status and insulin sensitivity is dependent on the adipocyte-specific peroxisome proliferator-activated receptor-gamma2 isoform. Diabetes 54: 1706-1716.

3. Burcelin R, Crivelli V, Dacosta A, Roy-Tirelli A, Thorens B (2002) Heterogeneous metabolic adaptation of C57BL/6J mice to high-fat diet. Am J Physiol Endocrinol etabol 282: E834-842.

4. Bohlooly YM, Olsson B, Bruder CE, Linden D, Sjogren K, et al. (2005) Growth hormone overexpression in the central nervous system results in hyperphagia-induced obesity associated with insulin resistance and dyslipidemia. Diabetes 54: 51-62.

5. McQueen AP, Zhang D, Hu P, Swenson L, Yang Y, et al. (2005) Contractile dysfunction in hypertrophied hearts with deficient insulin receptor signaling: possible role of reduced capillary density. J Mol Cell Cardiol 39: 882-892.

6. Lelliott CJ, Lopez M, Curtis RK, Parker N, Laudes M, et al. (2005) Transcript and metabolite analysis of the effects of tamoxifen in rat liver reveals inhibition of fatty acid synthesis in the presence of hepatic steatosis. FASEB J 19: 1108-1119.

7. Linden D, William-Olsson L, Ahnmark A, Ekroos K, Hallberg C, et al. (2006) Liver-directed overexpression of mitochondrial glycerol-3-phosphate acyltransferase results in hepatic steatosis, increased triacylglycerol secretion and reduced fatty acid oxidation. FASEB J 20: 434-443.

8. De Matteis R, Ricquier D, Cinti S (1998) TH-, NPY-, SP-, and CGRP-immunoreactive nerves in interscapular brown adipose tissue of adult rats acclimated at different temperatures: an immunohistochemical study. J Neurocytol 27: 877-886.

9. Howard CV, Reed MG (1998) Unbiased Stereology: Three Dimensional Measurement in Microscopy. Oxford: Bios Scientific Publishers.

10. Mattfeldt T, Mall G, Gharehbaghi H, Moller P (1990) Estimation of surface area and length with the orientator. J Microscopy 159: 301-317.

11. Saks VA, Veksler VI, Kuznetsov AV, Kay L, Sikk P, et al. (1998) Permeabilized cell and skinned fiber techniques in studies of mitochondrial function in vivo. Mol Cell Biochem 184: 81-100.

12. Leone TC, Lehman JJ, Finck BN, Schaeffer PJ, Wende AR, et al. (2005) PGC-1alpha deficiency causes multi-system energy metabolic derangements: muscle dysfunction, abnormal weight control and hepatic steatosis. PLoS Biol 3: e101.

13. Boudina S, Sena S, O'Neill BT, Tathireddy P, Young ME, et al. (2005) Reduced Mitochondrial Oxidative Capacity and Increased Mitochondrial Uncoupling Impair Myocardial Energetics in Obesity. Circulation 112: 2686-2695.

14. Rolfe DF, Hulbert AJ, Brand MD (1994) Characteristics of mitochondrial proton leak and control of oxidative phosphorylation in the major oxygen-consuming tissues of the rat. Biochim Biophys Acta 1188: 405-416.

15. Cadenas S, Echtay KS, Harper JA, Jekabsons MB, Buckingham JA, et al. (2002) The basal proton conductance of skeletal muscle mitochondria from transgenic mice overexpressing or lacking uncoupling protein-3. J Biol Chem 277: 2773-2778.

16. Echtay KS, Esteves TC, Pakay JL, Jekabsons MB, Lambert AJ, et al. (2003) A signalling role for 4-hydroxy-2-nonenal in regulation of mitochondrial uncoupling. EMBO J 22: 4103-4110.

17. Brand MD (1995) Measurement of mitochondrial protonmotive force. In: Brown GC, Cooper CE, editors. Bioenergetics - A Practical Approach. Oxford, UK: IRL Press. pp. 39-62.

18. Petalidis L, Bhattacharyya S, Morris GA, Collins VP, Freeman TC, et al. (2003) Global amplification of mRNA by template-switching PCR: linearity and application to microarray analysis. Nuc Acids Res 31: e142.

19. Curtis RK, Oresic M, Vidal-Puig A (2005) Pathways to the analysis of microarray data. Trends Biotechnol 23: 429-435.

20. Kanehisa M, Goto S, Kawashima S, Okuno Y, Hattori M (2004) The KEGG resource for deciphering the genome. Nuc Acids Res 32: D277-280.
