# Supplementary material for: Ablation of PGC-1β Results in Defective Mitochondrial Activity, Thermogenesis, Hepatic Function, and Cardiac Performance
Source: PLoS Biol. 2006 Nov 7;4(11):e369. doi: 10.1371/journal.pbio.0040369 (PMC1634886; doi:10.1371/journal.pbio.0040369)
Supplement: Supplementary file 3 [file pbio.0040369.st001.doc]

**Supplementary Table 1: WT and PGC1βKO mouse parameters for energy intake and faecal output over 48h**

|  | **Wild-type** | **PGC1βKO** | ***P*-value** |
| --- | --- | --- | --- |
| **Food Intake (g/48h)** | **8.48 ± 0.27** | **8.34 ± 0.27** | **NS** |
| **Body Weight (g)** | **29.99 ± 0.82** | **29.13 ± 0.71** | **NS** |
| **Food Intake/Body Weight (g)** | **0.29 ± 0.02** | **0.29 ± 0.01** | **NS** |
| **Energy In (Cal/48h)** | **156731 ± 4939** | **154097 ± 4976** | **NS** |
| **Faecal Energy Out (Cal/48h)** | **34459 ± 1412** | **34278 ± 1111** | **NS** |
| **Ratio (EI:EO)** | **29.99 ± 0.82** | **29.13 ± 0.71** | **NS** |

The food intake and energetic content of faeces from 12 week-old male mice was determined over a 48h period. *n*=5-8/group.
