# Supplementary material for: Ablation of PGC-1β Results in Defective Mitochondrial Activity, Thermogenesis, Hepatic Function, and Cardiac Performance
Source: PLoS Biol. 2006 Nov 7;4(11):e369. doi: 10.1371/journal.pbio.0040369 (PMC1634886; doi:10.1371/journal.pbio.0040369)
Supplement: Supplementary file 4 [file pbio.0040369.st002.doc]

**Supplementary Table 2: WT and PGC1βKO tissue weights in 14-week old male mice**

|  | **BAT (g)** | **WAT (g)** | **Liver (g)** | **Heart (g)** | **Total Soleus (g)** | **Total Gastro-cnemius (g)** | **Total Kidney (g)** | **Brain (g)** | **Total Testis (g)** |
| --- | --- | --- | --- | --- | --- | --- | --- | --- | --- |
| **WT** | 0.114 ± 0.012 | 0.42 ± 0.06 | 1.35 ± 0.10 | 0.166 ± 0.01 | 0.0132 ± 0.0012 | 0.294 ± 0.013 | 0.39 ± 0.01 | 0.453 ± 0.009 | 0.23 ± 0.02 |
| **PGC1βKO** | 0.140 ± 0.011 | 0.26 ± 0.02 | 1.43 ± 0.06 | 0.171 ± 0.00 | 0.0125 ± 0.0005 | 0.274 ± 0.007 | 0.38 ± 0.02 | 0.418 ± 0.014 | 0.23 ± 0.00 |
| ***P*-value (Raw weight)** | 0.16 | 0.02 | NS | NS | NS | 0.20 | NS | 0.05 | NS |
| ***P*-value (by body weight)** | 0.11 | 0.01 | 0.17 | NS | NS | NS | NS | 0.10 | NS |

The data is presented both comparing raw weights and as tissue weight/body weight. Statistical comparison between WT and PGC1βKO mice was performed using t-tests.  *n*=6-8/group. *P*-values are shown where *P* is less than or equal to 0.2
