# Supplementary material for: Ablation of PGC-1β Results in Defective Mitochondrial Activity, Thermogenesis, Hepatic Function, and Cardiac Performance
Source: PLoS Biol. 2006 Nov 7;4(11):e369. doi: 10.1371/journal.pbio.0040369 (PMC1634886; doi:10.1371/journal.pbio.0040369)
Supplement: Supplementary file 5 [file pbio.0040369.st003.doc]

**Supplementary Table 3: Comparison of KEGG pathways significantly downregulated in all 3 arrayed tissues (Liver, BAT and Heart)**

| **Pathway** | **Pathway Genes** | **BAT genes changed** | ***P*-value** | **Heart genes changed** | ***P*-value** | **Liver genes changed** | ***P*-value** |
| --- | --- | --- | --- | --- | --- | --- | --- |
| Oxidative Phosphorylation | 39 | 13 | 2.1x10^-11^ | 17 | 2.6x10^-14^ | 11 | 1.6x10^-15^ |
| ATP synthesis | 18 | 4 | 0.001 | 9 | 1.32x10^-8^ | 4 | 8.9x10^-7^ |

RNA was isolated from 14 week-old normal diet-fed male PGC1βKO and WT mice and then analysed using spotted arrays as described in the Materials and Methods. Data from these chips were then analysed using for pathway changes using the KEGG pathway database. Pathways with only 1 changed gene were discounted. Data is displayed as Pathway Name followed-by *P*-value for the binomial test. A pathway *P*-value of <0.05 was considered significant.
