# Supplementary material for: Ablation of PGC-1β Results in Defective Mitochondrial Activity, Thermogenesis, Hepatic Function, and Cardiac Performance
Source: PLoS Biol. 2006 Nov 7;4(11):e369. doi: 10.1371/journal.pbio.0040369 (PMC1634886; doi:10.1371/journal.pbio.0040369)
Supplement: Supplementary file 6 [file pbio.0040369.st004.doc]

**Supplementary Table 4: Sequences for Taqman primers and probes**

Sybr as a probe indicates the use of a Sybr-Green system instead of Taqman

| **Target** | **Forward primer** | **Reverse primer** | **Probe (5’ FAM, 3’ TAMRA)** |
| --- | --- | --- | --- |
| 36B4 | gaggaatcagatgaggatatggga | aagcaggctgacttggttgc | tcggtctcttcgactaatcccgccaa |
| Acyl-CoA oxidase (ACO1) | tgttgtccctatccgtgagattg | ggccgatatccccaacagt | acccacaagcctctgccaggca |
| Adiponectin (ACRP30) | tcctggagagaagggagagaaag | cagctcctgtcattccaacatc | agatgcaggtcttcttggtcctaagggtga |
| Adipose Differentiation Related Protein (ADRP) | gtagaccagtacttccctctcactca | ggcttctgaaccatatcaaatcct | caccttttttgcttccatctccagctcct |
| ATP synthase 6 (ATPase6) | ggctcccgacacaaactaaaaag | tggaattagtgaaattggagttcct | tcacttgcccacttccttcca |
| ATP synthase subunit β (ATPβ) | caccaccaagaagggatcga | gcagggtcagtcaggtcatca | acctcggtgcaggctatctatgtgcct |
| B-type natriuretic peptide (BNP) | gccagtctccagagcaattca | gggccatttcctccgactt | tgcagaagctgctggagctgataagaga |
| CCAAT/enhancer-binding protein-α (C/EBPα) | gagccgagataaagccaaaca | cggtcattgtcactggtcaact | aacgtggagacgcaacagaaggtgc |
| C/EBPβ | aagctgagcgacgagtacaaga | gtcagctccagcaccttgtg | cgcgagcgcaacaacatcgc |
| C/EBPδ | cgccgcaaccaggagat | gctgatgcagcttctcgttct | cagaagctggtggagttgtcggcc |
| Cardiac alpha actin (Cd a-actin) | ccagcccagctgaatcca | Acacatcctggcacagctttg | Sybr |
| Carnitine palmitoyltransferase 1a (CPT1a) | tgagtggcgtcctctttgg | cagcgagtagcgcatagtcatg | cagggctctgggtggcgatca |
| CPT1b | ccaaacgtcactgcctaagct | ggccgcacagaatccaagta | gccagcgtgccagccacaattc |
| Complex I, alpha-subcomplex 9 (α-s9) | cccgggccagcttacct | gctgcactgctttcctgataga | tctggaatgggatgcacgagacaaaga |
| Complex III, Cytochrome b subunit (Cytb) | gccaccttgacccgattct | ttgctagggccgcgataat | cggtttccacttcatcttaccatt |
| Complex III, Fe-S Core protein (Fe-S) | gctgggcgcacactttgt | cactggccttgcaggaagaa | tgtgatgccatgagtatcgatgacatggt |
| Cytochrome Oxidase subunit 1 (Cox 1) | ttttcaggcttcaccctagatga | gaagaatgttatgtttactcctacgaatatg | catgagcaaaagcccacttcgcca |
| Cox 2 | ccatcccaggccgactaaa | tttcagagcattggccatagaa | caagcaacagtaacatcaaaccgaccagg |
| Cox 3 | cggaagtatttttctttgcaggat | cagcagcctcctagatcatgtg | cttctgagcgttctatcattctagcctcgtacca |
