# Supplementary material for: Ablation of PGC-1β Results in Defective Mitochondrial Activity, Thermogenesis, Hepatic Function, and Cardiac Performance
Source: PLoS Biol. 2006 Nov 7;4(11):e369. doi: 10.1371/journal.pbio.0040369 (PMC1634886; doi:10.1371/journal.pbio.0040369)
Supplement: Supplementary file 7 [file pbio.0040369.st005.doc]

**Supplementary Table 5: Sequences for Taqman primers and probes**

Sybr as a probe indicates the use of a Sybr-Green system instead of Taqman

| **Target** | **Forward primer** | **Reverse primer** | **Probe (5’ FAM, 3’ TAMRA)** |
| --- | --- | --- | --- |
| Cox4 | tgcagaccaagcgaatgct | tagtcccacttggcggagaa | catgaaggccaaccccattcaggg |
| Cox5b | cgtccatcagcaacaagagaata | gcagccaaaaccagatgaca | tgggctgcatctgtgaagaggacaac |
| Creatine Kinase, muscle (CK) | aagagacaccatctggcttca | gaagaccgtgtaggactcctcatc | cttcatcatgacggtgggctgtgt |
| Cytochrome C (CytC) | ggctgctggattctcttacaca | ccaaatactccatcagggtatcct | ccaacaagaacaaaggcatcacctggg |
| Deiodinase 2 (D2) | tgcgctgtgtctggaacag | ctggaattgggagcatcttca | ttcctcctagatgcctacaaacaggttaaactgg |
| Fatty acid synthase (FAS) | cctggactcgctcatgggt | atttcctgaagtttccgcagc | cgtcagatcctggaacgagaacacga |
| Fatty acid-binding protein 4 (aP2) | gcgtggaattcgatgaaatca | cccgccatctagggttatga | cgcagacgacaggaaggtgaagagc |
| Glucose transporter 4 (GLUT4) | gattccatcccacaaggcac | tcatgccacccacagagaag | ctacgctctgggctctctccgtgg |
| Hormone sensitive lipase (HSL) | ggagcactacaaacgcaacga | tcggccaccggtaaagag | acaggcctcagtgtgaccgcca |
| Insulin receptor substrate 1 (IRS1) | gagcgcccccaaacg | gtcagcccgcttgttgatg | cgatccccctcgagagctgtttca |
| Insulin receptor substrate 2 (IRS2) | caagagccctggcgagtaca | ccgcggatgccagtagtg | caacattgactttggtgaggcaggtaccc |
| Isocitrate dehydrogenase 3, gamma (IDH) | ggttctggctgcccatgt | ccgcccaccatacttagca | ccccccgaaggagcatttcctca |
| Leptin | aaccctcatcaagaccattgtca | cctctgcttggaggatacc | caggatcaatgacatttcacacacgcag |
| Lipoprotein lipase (LPL) | gtggccgagagcgagaac | aagaaggagtaggttttatttgtggaa | ttcccttcaccctgcccgagg |
| MADS-box transcription enhancer factor 2c (MEF2c) | acataacatgccgccatctg | ggtgtgttgtgggtatctcgaa | cctcagtcagttgggagaccgtaccacc |
| Medium chain acyl-CoA dehydrogenase (MCAD) | tgacggagcagccaatga | atggccgccacatcaga | tgcttactgtgtgacagagccctccg |
| Mitochondrial Glycerol-3-phosphate acyltransferase (mGPAT) | ggcttgagagctgccacct | agccctttagctgttgggaag | tagtgcggccagtgactgcttcaaga |
| Mitochondrial Transcription Factor A (mtTFA) | ccacagaacagctacccaaattt | tccacagggctgcaattttc | aagctaaacacccagatgcaaaactttcagaatt |
| Myoglobin | ccagcctctagcccaatcac | atttctgagataaactccaggtacttga | cagctcctgtcattccaacatc |
