# Supplementary material for: Ablation of PGC-1β Results in Defective Mitochondrial Activity, Thermogenesis, Hepatic Function, and Cardiac Performance
Source: PLoS Biol. 2006 Nov 7;4(11):e369. doi: 10.1371/journal.pbio.0040369 (PMC1634886; doi:10.1371/journal.pbio.0040369)
Supplement: Supplementary file 8 [file pbio.0040369.st006.doc]

**Supplementary Table 6: Sequences for Taqman primers and probes**

Sybr as a probe indicates the use of a Sybr-Green system instead of Taqman

| **Target** | **Forward primer** | **Reverse primer** | **Probe (5’ FAM, 3’ TAMRA)** |
| --- | --- | --- | --- |
| NADH Dehydrogenase subunit 4 (ND4) | catcactcctattctgcctagcaa | tcctcgggccatgattatagtac | ctccaactacgaacggatccacagc |
| Nuclear Respiratory Factor 1 (NRF1) | aagagacagcagacacgtttgc | tcccactcgcgtcgtgtac | tcggaaactcagagccacgttggatg |
| Peroxisome proliferator-activated receptor-gamma coactivator-1, alpha (PGC-1α) | gtaggcccaggtacgacagc | gctctttgcggtattcatccc | atgaagcctatgagcacgaaaggctcaa |
| PGC-1β | ctccaggcaggttcaaccc | gggccagaagttcccttagg | ccccccaaagccttctggactgagt |
| Phosphoenolpyruvate carboxykinase (PEPCK) | ccacagctgctgcagaacac | gaagggtcgcatggcaaa | agggcaagatcatcatgcacgaccc |
| Peroxisome proliferator-activated receptor-α (PPARα) | cacgatgctgtcctccttga | gtgtgataaagccattgccgt | acaaagacgggatgctgatcgcg |
| PPARδ | ttcatcgcggccatcat | gtgtcctggatggcttctacct | ctgtgtggagaccggccaggc |
| PPARγ1 | tttaaaaacaagactaccctttactgaaatt | agaggtccacagagctgattcc | gagagatgccattctggcccac |
| PPARγ2 | gatgcactgcctatgagcactt | agaggtccacagagctgattcc | gagagatgccattctggcccac |
| Protein phosphatase 1 (PP1) | caaactcaacatcgacagcatca | cattcttgcctggcttgga | ccaacggctgctggaagtgagagg |
| Skeletal muscle actin (Sk actin) | cacccagggccagagtca | agagcggtggtctcgtcttc | Sybr |
| Stearoyl-CoA desaturase 1(SCD1) | cctgcggatcttccttatcatt | gatctcgggcccattcg | accatggcgttccagaatgacgtgt |
| Sterol response element-binding protein 1c (SREBP1c) | cacggagccatggattgc | cccgggaagtcactgtcttg | catttgaagacatgcatccagctcatcaaca |
| Succinate Dehydrogenase Subunit B (SDHB) | cgctgccacaccatcatg | tttccgcaatcgctttcc | acagacctgccccaagggtctg |
| Troponin T (TropT) | ctgcgaaaccggatcaatg | cgcccggtgactttgg | caaccagaaagtctccaaaactcgtggg |
| Uncoupling Protein 1 (UCP1) | ctgggcttaacgggtcctg | ctgggctaggtagtgccagtg | agctcaaaggtgagcgtcccttcctc |
| UCP2 | gacctcaaagcagcctccag | gagaaacggggaccttcaatc | tccggcagagttcctctgtctcgtctt |
| UCP3 | caaccttggctagacgcacag | tggaggtccgaggagagagc | tcctccctgaactgaagcaaaagattgcc |
| Uroporphyrinogen III synthase (Uro3) | ggagcctgaagagctactatgaaga | ggccggagggactgaaaa | cagggtatcccagccagcatcacg |
